# Burden of atrial fibrillation and atrial flutter attributable to smoking in the G20: Trends from 1990 to 2021 and predictions for 2022 to 2050: A secondary dataset analysis of the Global Burden of Disease (GBD) 2021

**DOI:** 10.18332/tid/216109

**Published:** 2026-03-18

**Authors:** Wanyue Yang, Guoping Ma, Hai Wang, Wenlu Zheng, Xuexin Cui, Ze Wang, Wenjie Liang

**Affiliations:** 1 Heart Center, Hebei Medical University First Affiliated Hospital, Shijiazhuang City, China; 2 College of Integrated Traditional Chinese and Western Medicine, Hebei Medical University College of Traditional Chinese Medicine, Shijiazhuang City, China

**Keywords:** atrial fibrillation, atrial flutter, smoking, burden of disease, G20

## Abstract

**INTRODUCTION:**

Atrial fibrillation and atrial flutter (AF/AFL) are major contributors to the cardiovascular disease burden in G20 countries. However, comprehensive assessments of smoking-attributable AF/AFL burden across the G20 remain limited. The objective of this study was to evaluate temporal trends in the AF/AFL burden attributable to smoking in G20 countries from 1990 to 2021 and to project future trends through 2050.

**METHODS:**

This secondary analysis used data from the Global Burden of Disease (GBD) 2021 study. Smoking-attributable disability-adjusted life years (DALYs), deaths, years lived with disability (YLDs), and years of life lost (YLLs) for AF/AFL were extracted for G20 countries between 1990 and 2021. Trends were analyzed by age, sex, and country, and estimated annual percentage changes (EAPCs) were calculated. Autoregressive integrated moving average (ARIMA) and exponential smoothing (ES) models were applied to project the disease burden from 2022 to 2050.

**RESULTS:**

In 2021, smoking-attributable AF/AFL accounted for 321761.89 DALYs (95% UI: 187788.89–476327.05) in the G20. Overall age-standardized rates remained relatively stable from 1990 to 2021. Japan showed a declining trend, whereas Saudi Arabia and Indonesia exhibited increasing burdens. Males consistently experienced higher burdens than females, and the highest absolute burden occurred in individuals aged 65–89 years. Projections indicate that from 2022 to 2050, absolute numbers of deaths, YLDs, YLLs, and DALYs attributable to smoking-related AF/AFL will continue to rise, particularly among males, despite stable or slightly declining age-standardized rates.

**CONCLUSIONS:**

Although age-standardized smoking-attributable AF/AFL rates in G20 countries have remained largely stable, absolute burdens are expected to increase substantially due to population growth and ageing. Pronounced sex- and country-level heterogeneity highlights the need for sustained and targeted tobacco control and cardiovascular prevention strategies.

## INTRODUCTION

Atrial fibrillation (AF) and atrial flutter (AFL) are the most common persistent arrhythmias and are closely associated with increased risks of stroke, heart failure, and all-cause mortality; thus, they are emerging as significant public health concerns^1-3^. Worldwide, there are 33.5 million patients with AF, with a prevalence comparable to that of myocardial infarction. The related medical burden continues to increase, and prevention and control strategies targeting modifiable risk factors are urgently needed^4-6^.

Studies have shown that smoking is closely related to AF/AFL. Mendelian randomization studies, as well as numerous large-scale clinical studies and meta-analyses, have indicated that current smoking, previous smoking, and light smoking all significantly increase the risk of AF/AFL in a dose-dependent manner^7-9^. The mechanisms of AF/AFL attributable to smoking include inducing myocardial fibrosis, disrupting atrial electrical stability, affecting ion channels, triggering cardiovascular diseases (CVDs), reducing lung function, etc.^8^. Smoking, an important regulatory risk factor for CVDs, has been proven to be directly related to various cardiovascular subtypes, such as ischemic heart disease and aortic aneurysm^10^. Previous studies have also revealed that smoking is an important risk factor affecting the G20 disease burden and mortality attributed to AF/AFL^6,11^. The G20 is an international economic cooperation forum that includes both developed and developing countries. The G20 represents most of the world’s largest economies and encompasses two-thirds of the G20 population^12,13^. These countries exhibit significant diversity in geography, economic development, healthcare systems, and medical resources. Although the global burden of atrial fibrillation and atrial flutter (AF/AFL) has been extensively reported, evidence regarding the smoking-attributable AF/AFL burden has largely focused on global or regional estimates, and comprehensive G20-specific assessments remain limited.

Therefore, this study aimed to systematically assess the burden of atrial fibrillation and atrial flutter (AF/AFL) attributable to smoking in G20 countries from 1990 to 2021, and to project future trends in the smoking-attributable AF/AFL burden from 2022 to 2050.

## METHODS

### Study design and time frame

This study was a secondary dataset analysis based on estimates from the Global Burden of Disease (GBD) Study 2021. We examined the burden of atrial fibrillation and atrial flutter (AF/AFL) attributable to smoking in G20 countries over the period from 1990 to 2021 and projected future trends from 2022 to 2050.

### Data sources

The data used in this study were derived from the GBD 2021 database, which uses epidemiological data and improved standardized methods; the GBD study conducts a comprehensive assessment of 371 diseases and injuries, as well as 88 risk factors, in 204 countries and regions. The GBD database is a powerful tool for analyzing epidemiological shifts^14,15^. The GBD study is a systematic, international collaborative initiative aimed at evaluating G20 health challenges and is led by the Institute for Health Metrics and Evaluation (IHME) in the United States. The GBD study integrates multisource data, including vital registration and health system data (e.g. death registry records, hospital records, and cancer registries), epidemiological survey and cohort study data (e.g. population health surveys and disease-specific surveillance), satellite and remote sensing data (e.g. environmental exposures and climate metrics), and data from novel sources (such as mobile health data), with continuous updates to ensure timeliness. The raw data are processed and calibrated using advanced statistical models, including the Cause of Death Ensemble model (CODEm) and DisMod-MR 2.1 (a Bayesian meta-regression model), to correct biases and predict missing values^16-18^. The G20 countries included in this study were Argentina, Australia, Brazil, Canada, China, France, Germany, India, Indonesia, Italy, Japan, Mexico, the Republic of Korea, the Russian Federation, Saudi Arabia, South Africa, Türkiye, the United Kingdom, the United States, and the European Union.

### Definitions of relevant indicators

The International Classification of Diseases and Injuries (ICD-9 and ICD-10) was used to identify patients with AF/AFL. All CVDs, encoded as 427.3-427.32 in the ICD-9 and I48-I48.92 in the ICD-10, were identified as AF/AFL in this study^6,19^. Similar to previous GBD studies^6,19^, an electrocardiogram was used to diagnose AF/AFL.

According to the GBD 2021 research standard, participants are classified into two groups based on their smoking behaviour: current smokers, who are currently consuming any form of tobacco products, and previous smokers, who have a history of smoking and have completely stopped using all tobacco products for six months or more^10,15^.

The calculation method of years of lost life (YLLs) is the standard life expectancy at the time of death multiplied by the number of deaths. Years lived with disabilities (YLDs) are estimated as years of life with disability weighted by the severity of disability. The sum of the number of years of death resulting from premature population deaths and the number of years of death is regarded as disability-adjusted life years (DALYs) ^6,11,20^. Age-standardization rates (ASRs) are determined by adjusting the global age distribution to the standard population. This adjustment is calculated as the weighted sum of specific age ratios, eliminating the confusion effect caused by age differences in the population, and thus enabling more precise comparative analysis of population groups^11,21^.

### Uncertainty intervals (UIs) and confidence intervals (CIs)

In this study, uncertainty intervals (UIs) and confidence intervals (CIs) are reported to reflect different sources of uncertainty. The 95% uncertainty intervals (UIs) provided by the Global Burden of Disease (GBD) Study are derived from repeated sampling of the posterior distribution of the model estimates and therefore capture uncertainty arising from multiple sources, including data availability, data quality, and model specification. In contrast, 95% confidence intervals (CIs) are generated using conventional statistical methods and reflect the sampling variability around point estimates, assuming a fixed underlying model. Reporting both measures helps improve transparency and interpretability for readers with different levels of statistical expertise.

### Forecasting analysis

Time-series forecasting of smoking-attributable atrial fibrillation and atrial flutter (AF/AFL) burden from 2022 to 2050 was conducted using autoregressive integrated moving average (ARIMA) and exponential smoothing (ES) models. Forecasts were based on annual time-series data from 1990 to 2021 at the G20 aggregate level. Separate models were fitted for each outcome indicator, including deaths, years lived with disability (YLDs), years of life lost (YLLs), and disability-adjusted life years (DALYs). In addition, sex-stratified time series were modeled to explore potential differences between males and females. For ARIMA modeling, stationarity of the time series was assessed visually and using the augmented Dickey–Fuller test. Appropriate differencing was applied when necessary to achieve stationarity. Model parameters were selected based on standard diagnostics and information criteria, including the Akaike information criterion (AIC).

Exponential smoothing models were selected based on the underlying trend characteristics of each time series, and the optimal ES specification was determined using goodness-of-fit measures. Model adequacy was evaluated through residual diagnostics and comparison of fitted versus observed values.

To assess internal validity, models were trained on data from an earlier period and their short-term forecasts were compared with subsequently observed values, ensuring reasonable predictive performance before extending the projections to 2050.

### Multiple comparisons and interpretation of uncertainty

No formal correction for multiple comparisons was applied in this study. Analyses were descriptive in nature and did not involve hypothesis testing based on p-values. Given the multiplicity and uncertainty inherent in age-standardized rates derived from Global Burden of Disease (GBD) posterior draws, differences between groups and over time were interpreted based on the direction and magnitude of estimated effects, as well as the overlap or non-overlap of uncertainty intervals (UIs), rather than formal statistical significance.

### Handling of missing data

This study was based on estimates extracted directly from the Global Burden of Disease (GBD) Study 2021. At the level of the extracted outcome measures (deaths, YLDs, YLLs, and DALYs), no missing data were encountered. Any missing or sparse primary input data in the original data sources were addressed within the GBD modeling framework through standardized data synthesis and estimation procedures. Therefore, no additional handling of missing data or imputation was performed in the present analysis.

### Statistical analysis

The estimated annual percentage change (EAPC) can serve as a concise measure for evaluating the trend of the ASR within a specific period^11^. The EAPC is derived using the formula 100 × [exp(β) - 1] (β is the slope of time), and its 95% confidence interval (CI) is determined through parameter estimates from linear regression analysis^21^. The following criteria are followed for trend interpretation: an upwards trend when the 95% CI remains entirely positive, a downwards trend when it is entirely negative, and stability when it includes zero^22^.

In this study, we extracted detailed data on the indicators of AF/AFL caused by smoking, including DALYs, deaths, YLDs, and YLLs, for in-depth analysis. First, we analyzed the number of cases of AF/AFL caused by smoking worldwide from 1990 to 2021 and the corresponding ASRs to investigate the dynamic trends in the disease burden. We subsequently evaluated subgroup differences by stratifying the 2021 data by sex, age group, and G20 country. Further analyses were conducted at the G20 country level to descriptively examine temporal patterns and heterogeneity in smoking-attributable AF/AFL burden. No formal decomposition analysis (e.g. Das Gupta method) was performed, and changes were evaluated based on trends in absolute numbers, age-standardized rates, and EAPCs. Finally, we used the autoregressive integral moving average (ARIMA) model and the exponential smoothing (ES) model to predict the burden of AF/AFL from 2022 to 2050. Differences between sexes, age groups, and countries were assessed descriptively by comparing temporal trends in absolute numbers, age-standardized rates (ASRs), and estimated annual percentage changes (EAPCs), rather than through formal statistical tests of trend differences between groups. All analyses and graphing were completed using R statistical software (version 4.4.1), and statistical significance was set at p<0.05.

## RESULTS

### Disease burden and development trend of AF/AFL caused by smoking in G20 countries from 1990 to 2021

**Figure 1:**
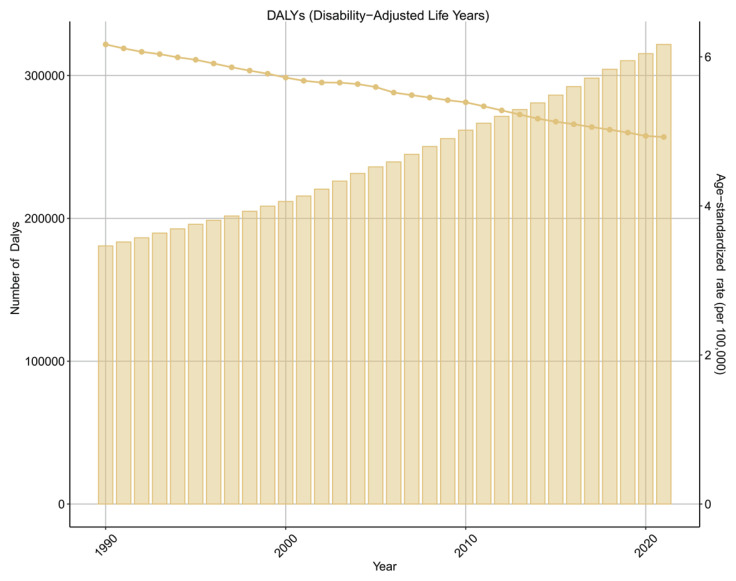
Disability-adjusted life years (DALYs) and age-standardized DALY rates of atrial fibrillation and atrial flutter attributable to smoking in G20 countries, 1990–2021

From 1990 to 2021, the absolute numbers of deaths, YLDs, YLLs, and DALYs attributable to smoking-related AF/AFL increased steadily across G20 countries. However, the corresponding age-standardized rates exhibited relatively stable trends, as reflected by small or non-significant estimated annual percentage changes (EAPCs). The disease burden of AF/AFL caused by smoking showed a stable trend. The EAPCs of DALYs, deaths, YLDs and YLLs for AF/AFL caused by smoking were 0.12 (95% CI: -0.09–0.33), 0.31 (95% CI: 0–0.62), 0.14 (95% CI: -0.06–0.35) and 0.09 (95% CI: -0.15–0.33), respectively (Figure 1 and Table 1; and Supplementary file Figures S1–S3 and Tables S1–S3).

**Table 1:** Trends in disability-adjusted life years (DALYs) attributable to smoking-related atrial fibrillation and atrial flutter (AF/AFL) in G20 countries from 1990 to 2021, including the number of DALYs, age-standardized DALY rates (per 100000 population), and estimated annual percentage changes (EAPCs), based on data from the Global Burden of Disease Study 2021

*G20 Countries*	*1990*		*2021*		*EAPC (95% CI)*
	*Number of DALYs* *(95% UI)*	*The age-standardized DALYs rate/100000* *(95% UI)*	*Number of DALYs* *(95% UI)*	*The age-standardized DALYs rate/100000* *(95% UI)*	
	180713.02 (106284.63–271001.33)	6.17 (3.63–9.29)	321761.89 (187788.89–476327.05)	4.92 (2.88–7.3)	0.12 (-0.09–0.33)
**Argentina**	1246.33 (734.82–1886.98)	3.87 (2.28–5.86)	1610.75 (936.01–2436.15)	2.94 (1.72-4.46)	-0.22 (-0.41 – -0.03)
**Australia**	1477.71 (869.74–2200.1)	7.67 (4.51–11.39)	1893.61 (1051.68–2978.27)	4.44 (2.45–6.92)	-1.04 (-1.51 – -0.57)
**Brazil**	7576.43 (4379.7–11451.34)	8.87 (5.18–13.35)	12118.31 (6857.95–18679.46)	4.82 (2.73–7.45)	-1.01 (-1.42 – -0.6)
**Canada**	3236.03 (1842.99–5033.19)	9.96 (5.73–15.43)	3864.82 (2136.83–6162.9)	5.4 (3.01–8.46)	-1.26 (-1.84 – -0.67)
**China**	44181.87 (26151.96–65797.25)	6.19 (3.66–9.19)	113500.61 (63199.64–171028.24)	5.51 (3.05–8.26)	1.2 (0.79–1.61)
**European Union**	45395.14 (26432.87–69161.39)	7.74 (4.53–11.74)	56779.6 (32744.22–86186.78)	6.3 (3.69–9.4)	0 (-0.71–0.71)
**France**	5754.72 (3180.41–8843.8)	7.12 (3.98–10.77)	6825.21 (3879.42–10613.69)	5.36 (3.02–8.25)	-0.4 (-1.07–0.28)
**Germany**	11061.31 (6319.5–16860.48)	8.93 (5.1–13.53)	15929.63 (9137.62–24424.48)	8.93 (5.16–13.64)	0.74 (-0.08–1.57)
**India**	16021 (8644.15–24697.13)	3.95 (2.12–6.06)	34760.97 (19795.27–53021.16)	3.22 (1.85–4.91)	0.19 (-0.57–0.96)
**Indonesia**	5285.77 (3038.87–7913.35)	5.93 (3.43–8.96)	15629.41 (8886.78–23307.93)	7.43 (4.26–11.05)	1.4 (0.64–2.16)
**Italy**	6327.05 (3565.49–9766.02)	7.36 (4.16–11.26)	6702.12 (3706.77–10608.26)	4.84 (2.69–7.75)	-0.65 (-1.49–0.19)
**Japan**	11389.39 (6777.31–16963.73)	6.71 (3.98–9.97)	10597.89 (6053.41–16483.91)	3.46 (1.97–5.25)	-1.77 (-2.64 – -0.89)
**Mexico**	2720.91 (1613.64–4051.32)	7.16 (4.2–10.61)	4596.89 (2616.08–7140.52)	3.68 (2.11–5.75)	-1.07 (-1.74 – -0.39)
**Republic of Korea**	2147.72 (1229.16–3289.74)	7.15 (4.12–10.87)	5102.89 (2945.62–7752.28)	5.67 (3.3–8.56)	0.81 (0.39–1.23)
**Russian Federation**	7849.78 (4410.88–11862.34)	4.32 (2.42–6.49)	12985.58 (7782.53–19300.47)	5.53 (3.29–8.28)	1.33 (0.82–1.84)
**Saudi Arabia**	66.53 (35.89–106.02)	1.06 (0.58–1.64)	370.8 (200.79–567.85)	1.5 (0.84–2.25)	1.95 (0.94–2.97)
**South Africa**	994.63 (571.59–1476.36)	4.94 (2.84–7.42)	1387.15 (820.63–2076.32)	2.94 (1.75–4.42)	-1.12 (-1.72 – -0.51)
**Turkey**	1261.94 (718.23–1984.58)	3.72 (2.09–5.76)	2325.02 (1321–3446.32)	2.53 (1.43–3.74)	-0.57 (-0.99 – -0.14)
**United Kingdom**	7936.63 (4610.94–12057.55)	8.74 (5.1–13.21)	7377.06 (4184.2–11287.88)	5.62 (3.19–8.69)	-1.2 (-1.93 – -0.46)
**United States**	21925.21 (12394.64–34060.37)	7 (3.97–10.77)	36860.55 (21247.23–55370.31)	6.37 (3.7–9.49)	0.25 (-0.24–0.74)

UI: uncertainty interval. CI: confidence interval.

**Figure 2:**
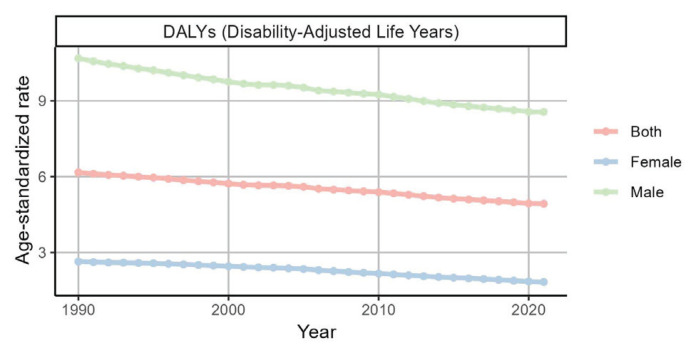
Trends in age-standardized DALY rates of atrial fibrillation and atrial flutter attributable to smoking in G20 countries by sex, 1990–2021

We further analyzed the effect of smoking on AF/AFL from a sex perspective. Over time, the burden of AF/AFL attributable to smoking in men was much greater than that in women, and the rates of DAYLs, deaths, YLDs, and YLLs in men were all greater than those in women (Figure 2 and Table 2; and Supplementary file Figures S4–S10 and Tables S4–S6).

**Table 2:** Trends in disability-adjusted life years (DALYs) attributable to smoking-related atrial fibrillation and atrial flutter (AF/AFL) stratified by sex and age group in G20 countries from 1990 to 2021, including the number of DALYs, age-standardized DALY rates (per 100000 population), and estimated annual percentage changes (EAPCs), based on data from the G20 Burden of Disease Study 2021

	*1990*		*2021*		*EAPC (95% CI)*
	*Number of DALYs* *(95% UI)*	*Age-standardized DALYs rate/100000* *(95% UI)*	*Number of DALYs* *(95% UI)*	*Age-standardized DALYs rate/100000* *(95% UI)*	
**Sex**					
Both	180713.02 (106284.63–271001.33)	6.17 (3.63–9.29)	321761.89 (187788.89–476327.05)	4.92 (2.88-7.3)	-0.72 (-0.74 – -0.71)
Female	41761.66 (24059.65–63323.28)	2.64 (1.51–3.99)	65856.23 (37165–100291.53)	1.83 (1.04–2.79)	-1.22 (-1.29 – -1.15)
Male	138951.36 (81538.52–206250.99)	10.68 (6.32–15.89)	255905.66 (150405.56–376207.78)	8.56 (4.99–12.63)	-0.7 (-0.72 – -0.68)
**Age** (years)					
30–34	790.45 (453.55–1237.72)	0.29 (0.16–0.45)	823.62 (457.39–1257.6)	0.21 (0.12–0.32)	-0.86 (-0.94 – -0.77)
35–39	2688.26 (1343.17–4965.03)	1.03 (0.51–1.9)	2836.97 (1455.26–5034.3)	0.78 (0.4–1.38)	-0.98 (-1.06 – -0.89)
40–44	6168.43 (3402.96–10632.84)	2.86 (1.58–4.93)	7126.13 (3859.52–12156.8)	2.16 (1.17–3.69)	-0.96 (-1.08 – -0.84)
45–49	9762.2 (5370.81–16044.14)	5.65 (3.11–9.28)	14139.36 (7555.51–23281.72)	4.3 (2.3–7.08)	-0.83 (-0.97 – -0.69)
50–54	15543.45 (8749.89–24237.52)	9.77 (5.5–15.23)	24973.84 (13733.16–39967.36)	7.78 (4.28–12.45)	-0.66 (-0.75 – -0.56)
55–59	20690.9 (11651.01–31637.64)	14.67 (8.26–22.43)	36084.03 (20359.68–55224.42)	12.39 (6.99–18.96)	-0.41 (-0.47 – -0.35)
60–64	25338.11 (14156.22–38593.21)	20.59 (11.51–31.37)	39491.93 (22043.29–61048.46)	16.81 (9.39–25.99)	-0.56 (-0.6 – -0.51)
65–69	26408.21 (15103.74–40712.29)	27.5 (15.73–42.39)	46107.5 (26587.59–70710.54)	21.77 (12.55–33.38)	-0.72 (-0.78 – -0.67)
70–74	23868.84 (13390.32–37220.04)	36.38 (20.41–56.72)	46539.52 (26218.08–72390.53)	28.72 (16.18–44.68)	-0.81 (-0.85 – -0.76)
75–79	21321.99 (11762.23–33478.71)	43.69 (24.1–68.61)	36612 (20298.13–57559.39)	35.24 (19.54–55.41)	-0.76 (-0.79 – -0.74)
80–84	15091.45 (8302.52–23548.5)	53.49 (29.43–83.47)	30168.73 (16709.25–48015.23)	43.15 (23.9–68.68)	-0.77 (-0.82 – -0.72)
85–89	8503.67 (4724.24–13095.34)	71.07 (39.48–109.44)	21025.23 (11690.06–32891.25)	56.45 (31.38–88.3)	-0.84 (-0.91 – -0.77)
90–94	3651.08 (2025.61–5537.68)	107.29 (59.52–162.73)	12168.9 (6491.94–18636.33)	82.36 (43.94–126.13)	-0.95 (-1.02 – -0.89)
≥95	886 (471.84–1348.5)	111.93 (59.61–170.36)	3664.13 (1927.41–5698.27)	82.17 (43.23–127.79)	-1.13 (-1.27 – -1.00)

UI: uncertainty interval. CI: confidence interval.

**Figure 3:**
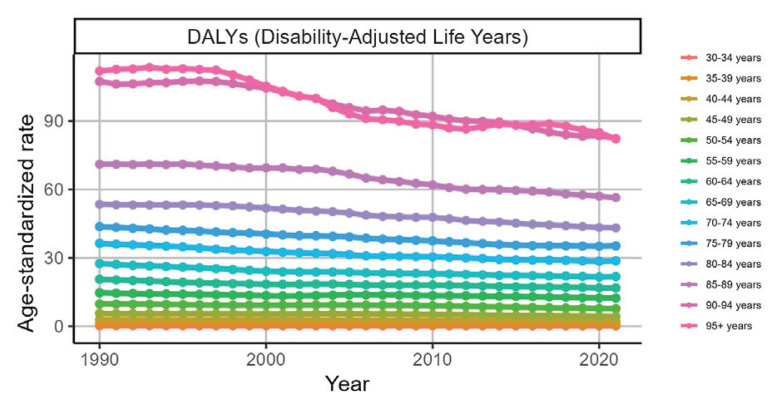
Trends in age-standardized DALY rates of atrial fibrillation and atrial flutter attributable to smoking across age groups in G20 countries, 1990–2021

The disease burden also varied greatly among different age groups. The ASR of the group aged ≥90 years was all at the top, whereas the burden of the group aged 35–75 years changed little over time and was almost flat. From the perspective of the number of cases of AF/AFL related to the three factors in the age group 70–89 years was relatively high, whereas in the age group of 33–44 years, it tended to change at a level close to the horizontal axis (Figure 3 and Table 2; and Supplementary file Figures S11–S17 and Tables S4–S6).

**Figure 4:**
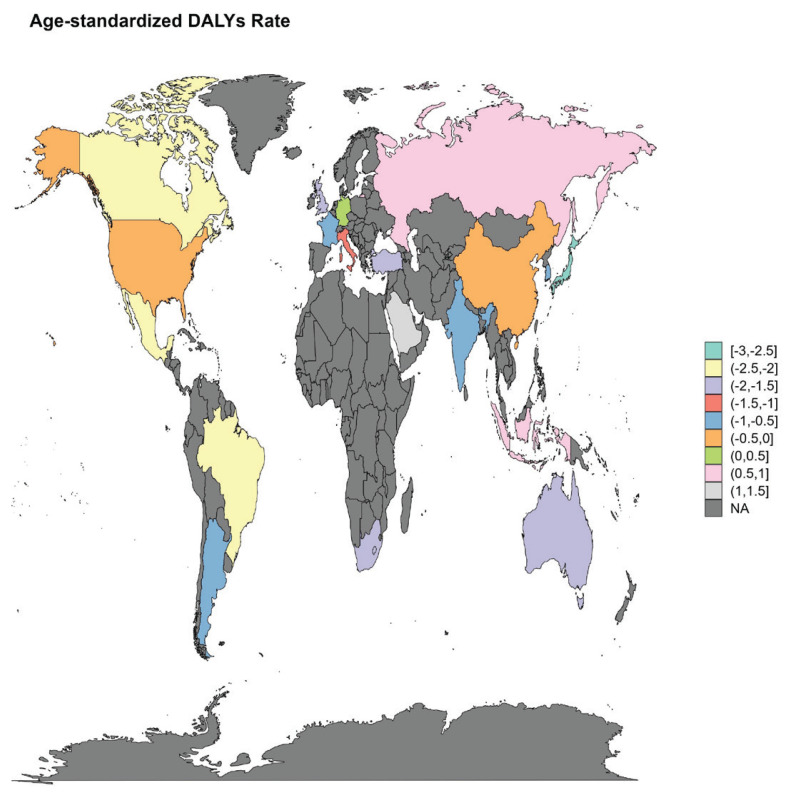
Global distribution of age-standardized DALY rates of atrial fibrillation and atrial flutter attributable to smoking, 2021

We conducted stratified analyses to examine changes in the AF/AFL burden across G20 countries. For AF/AFL caused by smoking, the trend of burden change in Japan decreased most significantly; the EAPCs of DALYs, deaths, YLDs and YLLs were -1.77 (95% CI: -2.64 – -0.89), -1.84 (95% CI: -2.99 – -0.69), -1.59 (95% CI: -2.38 – -0.79) and -1.99 (95% CI: -2.97 – -1.00), respectively; and the burden trend increased most significantly in Saudi Arabia and Indonesia (Figure 4 and Table 1; and Supplementary file Figures S18–S24 and Tables S1–S3).

### The 2021 disease burden of AF/AFL caused by smoking in the G20

We conducted a detailed analysis of the disease burden caused by smoking in 2021 to understand the latest burden of AF/AFL. Within the G20, the burdens of DALYs, deaths, YLDs, and YLLs for smoking-related AF/AFL in 2021 were 321761.89 (95% UI: 187788.89–476327.05), 8102.87(95% UI: 4749.03–11785.58), 189297.95 (95% UI: 102821.7–303660.85), and 132463.93 (95% UI: 78787.59–189831.89) , respectively. The ASRs of age were 4.92 (95% UI: 2.88–7.3), 0.13 (95% UI: 0.07–0.18), 2.88 (95% UI: 1.57–4.61) and 2.04 (95% UI: 1.22–2.93) (Table 1; and Supplementary file Tables S1–S3).

First, from the perspective of sex, in 2021, in terms of smoking-related AF/AFL, the burden was significantly greater in men than in women, and the burden was three times greater in men than in women (Table 2; and Supplementary file Figure S25 and Tables S4–S6). From the perspective of age subgroups, the ASRs of deaths, YLLs, and DALYs increased with increasing age. The group aged 75–79 years had the maximum values of smoking-related AF/AFL in terms of YLDs (19.11; 95% UI: 9.92–33.14). The numbers of DALYs, deaths, YLDs, and YLLs first increased, then decreased with age, and the peaks differed; however, all were within the age range of 65–89 years (Table 2; and Supplementary file Figure S26 and Tables S4–S6). The changes in sex and age-related burdens also indicated that men are at greater risk than women are, and the risk is more prominent for men in middle and old age (Supplementary file Figure S27).

In addition, the burden differences within G20 countries were also analyzed. China was the region with the highest number of cases of AF/AFL caused by smoking. The number of DALYs, deaths, YLDs and YLLs were 113500.61 (95% UI: 63199.64–171028.24), 2681.73 (95% UI: 1472.27–4093.2), and 71233.57 (95% UI: 37161.84–114970.05, 42267.04 (95% UI: 23274.84–64014.8), respectively. Saudi Arabia had the fewest, the number of DALYs, deaths, YLDs, and YLLs were 370.8 (95% UI: 200.79–567.85), 5.16 (95% UI: 2.67–8.05), 208.28 (95% UI: 96.93–356.8), and 162.52 (95% UI: 83.58–256.19), respectively. (Table 1; and Supplementary file Figure S28 and Tables S1–S3).

### Results of the prediction of the disease burden of AF/AFL caused by smoking in G20 countries from 2022 to 2050

**Figure 5:**
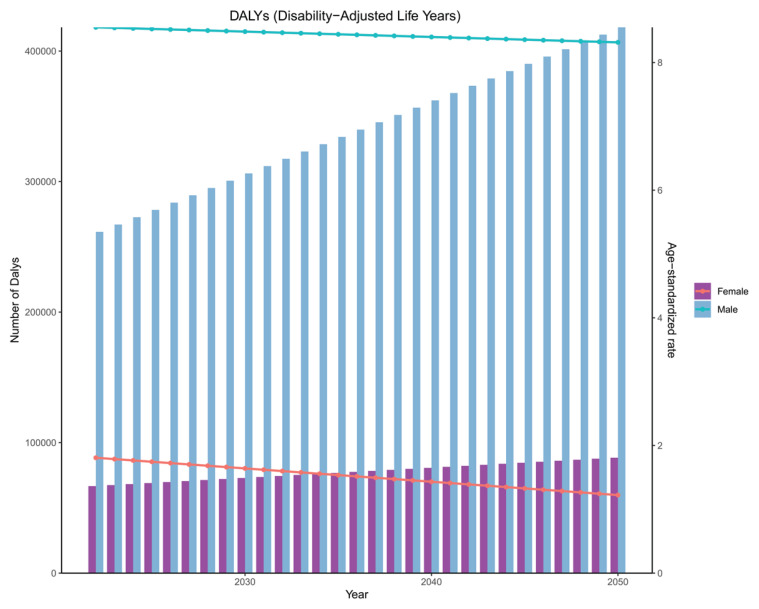
Projected trends in disability-adjusted life years (DALYs) and age-standardized DALY rates of atrial fibrillation and atrial flutter attributable to smoking in G20 countries by sex, 2022–2050

To guide the formulation of AF/AFL prevention and treatment strategies, we predicted the disease burden in the coming decades. For AF/AFL caused by smoking, the results of the two models [ARIMA (Figure 5; and Supplementary file Figures S29–S31) and ES (Supplementary file Figure S32)] were similar. Specifically, the number increased, and the burden on men was significantly greater than that on women. Except for YLDs, the slopes of the ASR changes were all relatively small and showed a downward trend.

## DISCUSSION

This study conducted a comprehensive analysis of the disease burden of AF/AFL caused by smoking within the G20 from 1990 to 2021. The disease burden of AF/AFL caused by smoking tended to be stable. In recent years, the prevalence of smoking has become an important factor in G20 mortality. Previous studies have shown that although deaths caused by smoking-related CVDs and the ASR of YLDs have decreased globally and in all GBD regions, the overall burden of smoking-related CVDs continues to increase^10^. Studies have shown that smokers have a greater risk of CVDs than non-smokers^23,24^. Some G20 countries have implemented strict tobacco control legislation, which has reduced smoking rates, resulting in a significant and sustained decline in per capita tobacco consumption, as seen in Australia, Japan, and Brazil^25^. These relevant policies have led to a decrease in tobacco consumption, thereby indirectly resulting in no significant increase in the disease burden of smoking-related AF/AFL.

The coexistence of increasing absolute numbers and relatively stable age-standardized rates suggests that population growth and population ageing, rather than an increase in age-specific risk, are the primary drivers of the observed rise in the overall burden of smoking-related AF/AFL in G20 countries. This difference may be due to many factors, including physiological factors, lifestyle, and disease management. Moreover, the proportion and amount of smoking among men are usually greater than those among women, highlighting the significant effect of unhealthy living habits on diseases in men. This further increases the burden of smoking-related AF/AFL among men^10^. The sex differences in the burden of disease may be related not only to the prevalence of smoking but also to the age at which smoking began and the differences in hormones^10^. A risk analysis of the age at smoking initiation revealed that for each year of delay in smoking initiation, the risk of cardiovascular events decreased by 4%. Furthermore, the age at which men start smoking is usually younger than that of women^26,27^. In addition to the age at smoking initiation, hormones also play a key role in regulating cardiovascular function. Estrogen can reduce the occurrence of cardiovascular events by improving vasodilation, regulating lipid metabolism, and exerting anti-atherosclerotic effects^28^. Studies have shown that the incidence of AF in premenopausal women is relatively low, but it increases after menopause, especially after 50 years of age. These findings indicate that estrogen may have a protective effect and that a decrease in estrogen levels after menopause may be harmful. A decrease in estrogen levels can lead to elevated blood pressure, high low-density lipoprotein cholesterol, metabolic syndrome, and increased BMI, all of which are risk factors for AF^29^. These factors are crucial for explaining the sex differences in the distribution of CVDs caused by smoking. Age analysis revealed that the burden of AF/AFL attributable to smoking was greater in the elderly group than in the young group. This might be related to the higher incidence of AF/AFL among the elderly population. This finding is relatively consistent with the burden of CVD onset on a global scale^10^.

The trends of the disease burden of AF/AFL caused by smoking varied from country to country and were influenced by local population factors, smoking rates, and tobacco control policies. The trend of burden changes in Japan is the most optimistic, which may be related to country-specific factors^25^. Australia has enacted smoke-free laws in all states and territories, increased taxes on tobacco products, included health warnings on tobacco products, and banned tobacco advertising and e-cigarette use. These efforts have led to a significant and sustained decrease in the smoking rate in Australia (from 43.4% in 2001 to 12.8% in 2017)^25,30^. The heavy disease burden in China is caused mainly by its large population^10^.

A higher smoking-attributable AF/AFL disease burden was observed among men, which may be related to differences in smoking prevalence and cumulative exposure between sexes. These projections suggest that the burden of atrial fibrillation and atrial flutter attributable to smoking may remain an important public health challenge in G20 countries over the coming decades. While the projected trends describe potential future trajectories rather than precise forecasts, they highlight the potential value of strengthened prevention efforts and sustained tobacco control measures to mitigate the smoking-attributable AF/AFL burden.

### Limitations

Several limitations of this study should be acknowledged. First, the estimates were derived from the Global Burden of Disease (GBD) 2021 framework and reflect population-level smoking-attributable burden rather than individual-level exposure or incident risk. As such, the observed associations are ecological in nature and should not be interpreted as causal relationships.

Second, this analysis focused on active smoking and did not account for secondhand smoking exposure, which may also contribute to the burden of atrial fibrillation and atrial flutter but could not be separately evaluated due to data limitations. In addition, potential interactions between smoking and other environmental, occupational, or behavioral risk factors were not explicitly modeled.

Third, estimated annual percentage changes (EAPCs) were calculated based on age-standardized rates and assume log-linear trends over time, which may not fully capture complex or non-linear temporal patterns.

Finally, future projections were generated using autoregressive integrated moving average (ARIMA) and exponential smoothing models, which rely on historical trends and underlying model assumptions. These projections are intended to illustrate potential future trajectories rather than to provide precise forecasts, and they may be affected by unforeseen changes in population structure, smoking behaviors, or public health interventions.

## CONCLUSIONS

Over the past 30 years, smoking has had a certain effect on the AF/AFL burden in the G20, but no significant increasing trend has been observed. The future disease burden remains substantial, with significant heterogeneity in the AF/AFL burden across sexes, ages, and nations. These projections highlight the potential value of context-specific preventive strategies to address the smoking-attributable AF/AFL burden.

## Supplementary Material



## Data Availability

The data supporting this research are available from the Global Burden of Disease Study 2021 (GBD 2021) at https://vizhub.healthdata.org/gbd-results/
