# Supplementary material for: Burden of atrial fibrillation and atrial flutter attributable to smoking in the G20: Trends from 1990 to 2021 and predictions for 2022 to 2050: A secondary dataset analysis of the Global Burden of Disease (GBD) 2021
Source: Tob Induc Dis. 2026 Mar 18;24:10.18332/tid/216109. doi: 10.18332/tid/216109 (PMC13005605; doi:10.18332/tid/216109)
Supplement: Supplementary file 1 [file TID-24-37-s1.pdf]

Table S1 Trends in deaths attributable to smoking-related atrial fibrillation and atrial flutter (AF/AFL) in G20 countries from 1990 to 2021, including the number of deaths, age-standardized death rates (per 100,000 population), and estimated annual percentage changes (EAPCs), based on data from the Global Burden of Disease Study 2021

	Deaths				
	Number of Deaths cases (95% UI) in 1990	The age-standardized Deaths rate/100000 (95% UI) in 1990	Number of Deaths cases (95% UI) in 2021	The age-standardized Deaths rate/100000 (95% UI) in 2021	EAPC (95% CI)
G20	3910.44 (2303.89-5614.18)	0.16 (0.09-0.23)	8102.87 (4749.03-11785.58)	0.13 (0.07-0.18)	0.31 (0-0.62)
Argentina	24.07 (14.57-36.33)	0.08 (0.05-0.12)	40.12 (23.28-62.46)	0.07 (0.04-0.11)	0.87 (0.52-1.23)
Australia	31.03 (18.5-46.12)	0.17 (0.1-0.25)	51.78 (28.27-80.67)	0.1 (0.06-0.15)	-0.52 (-1.16-0.12)
Brazil	142.45 (86.5-207.22)	0.23 (0.13-0.35)	285.7 (167.14-430.58)	0.12 (0.07-0.18)	-0.82 (-1.47--0.17)
Canada	67.55 (39.66-100.09)	0.21 (0.12-0.32)	96.41 (53.25-149.45)	0.12 (0.07-0.18)	-1.14 (-1.82--0.46)
China	935.68 (521.47-1434.38)	0.21 (0.12-0.33)	2681.73 (1472.27-4093.2)	0.16 (0.09-0.25)	0.8 (0.01-1.61)
European Union	1069.81 (641.01-1579.39)	0.18 (0.11-0.27)	1545.96 (900.71-2319.07)	0.13 (0.08-0.2)	0.29 (-0.59-1.18)
France	148.67 (86.25-230.25)	0.17 (0.1-0.26)	190.91 (104.39-298.68)	0.11 (0.06-0.17)	-0.52 (-1.45-0.42)
Germany	275.49 (154.09-409.06)	0.21 (0.12-0.31)	468.58 (263.84-715.93)	0.2 (0.11-0.3)	1.37 (0.34-2.42)
India	285.1 (136.26-468.67)	0.09 (0.05-0.16)	942.79 (530.27-1429.82)	0.11 (0.06-0.16)	1.59 (0.46-2.73)
Indonesia	88.73 (50.45-137.06)	0.15 (0.08-0.24)	315.42 (179.49-486.44)	0.25 (0.13-0.39)	2.16 (0.85-3.49)
Italy	118.65 (68.56-175.46)	0.14 (0.08-0.21)	180.67 (99.87-280.61)	0.1 (0.06-0.15)	0.31 (-0.79-1.43)
Japan	255.34 (158.07-367.26)	0.16 (0.1-0.24)	340.57 (194.72-513.04)	0.08 (0.04-0.11)	-1.84 (-2.99--0.69)
Mexico	69.87 (41.45-102.8)	0.25 (0.14-0.37)	112.66 (66.51-166.23)	0.1 (0.06-0.15)	-1.56 (-2.47--0.64)
Republic of Korea	36 (20.96-57.63)	0.18 (0.1-0.29)	129.01 (67.72-207.66)	0.14 (0.07-0.23)	1.56 (0.96-2.16)
Russian Federation	117.8 (66.19-176.43)	0.07 (0.04-0.11)	243.8 (149.85-354.82)	0.1 (0.06-0.15)	1.83 (1.35-2.32)
Saudi Arabia	1.22 (0.67-1.99)	0.03 (0.01-0.04)	5.16 (2.67-8.05)	0.03 (0.02-0.05)	1.26 (-0.24-2.78)

South Africa	21.14 (12.2-32.28)	0.13 (0.07-0.2)	28.8 (16.54-42.9)	0.08 (0.04-0.12)	-1.31 (-2.19--0.43)
Turkey	31.1 (16.98-50.23)	0.11 (0.06-0.18)	64.75 (36.45-100.5)	0.08 (0.04-0.12)	-0.15 (-0.76-0.46)
United Kingdom	267.23 (162.29-390.92)	0.28 (0.17-0.41)	278.51 (157.35-441.42)	0.18 (0.1-0.28)	-0.86 (-1.77-0.06)
United States of America	466.3 (275.67-697.75)	0.14 (0.08-0.21)	939.69 (536.42-1438.74)	0.15 (0.09-0.23)	0.7 (0.1-1.3)

Note: Data are presented as number of cases (95% uncertainty interval) and age-standardized rates per 100,000 population (95% UI). EAPC = estimated annual percentage change.

Table S2 Trends in years lived with disability (YLDs) attributable to smoking-related atrial fibrillation and atrial flutter (AF/AFL) in G20 countries from 1990 to 2021, including the number of YLD cases, age-standardized YLD rates (per 100,000 population), and estimated annual percentage changes (EAPCs), based on data from the Global Burden of Disease Study 2021

	YLDs				
	Number of YLDs cases (95% UI) in 1990	The age-standardized YLDs rate/100000 (95% UI) in 1990	Number of YLDs cases (95% UI) in 2021	The age-standardized YLDs rate/100000 (95% UI) in 2021	EAPC (95% CI)
G20	108216.23 (57638.88-176505.38)	3.54 (1.88-5.74)	189297.95 (102821.7-303660.85)	2.88 (1.57-4.61)	0.14 (-0.06-0.35)
Argentina	738.88 (362.74-1225.57)	2.26 (1.11-3.76)	862.72 (465.81-1467.2)	1.6 (0.86-2.72)	-0.86 (-1.02--0.69)
Australia	917.52 (510.67-1485.15)	4.76 (2.65-7.66)	1109.27 (568.63-1849.8)	2.77 (1.41-4.66)	-1.13 (-1.55--0.72)
Brazil	4798.49 (2455.98-7820.58)	5.25 (2.74-8.53)	7097.96 (3562.68-11675.34)	2.78 (1.4-4.58)	-1.07 (-1.42--0.72)
Canada	2114.88 (1098.45-3570.63)	6.48 (3.38-10.89)	2434.27 (1224.49-4122.56)	3.5 (1.77-5.87)	-1.25 (-1.81--0.68)
China	26200 (13621.74-41347.27)	3.15 (1.66-5)	71233.57 (37161.84-114970.05)	3.27 (1.72-5.31)	1.71 (1.36-2.07)
European Union	26794.3 (14243.31-44076.17)	4.59 (2.45-7.45)	33440.65 (18174.89-54353.49)	3.98 (2.16-6.39)	-0.09 (-0.74-0.57)

France	3368.08 (1716-5613.52)	4.29 (2.19-7.06)	3942.01 (2034.51-6745.57)	3.37 (1.72-5.67)	-0.42 (-1.03-0.18)
Germany	6437.29 (3288.6-10683.19)	5.3 (2.7-8.7)	9173.25 (4974.21-14769.62)	5.68 (3.07-9.26)	0.48 (-0.28-1.25)
India	9763.16 (5249.07-15891.08)	2.33 (1.25-3.85)	17706.03 (9162.3-29809.71)	1.56 (0.8-2.64)	-0.48 (-1.18-0.22)
Indonesia	3461.88 (1826.17-5538.85)	3.64 (1.92-5.8)	9637.55 (5079.11-15612.1)	3.96 (2.09-6.38)	1.09 (0.46-1.73)
Italy	4278.92 (2220.56-7077.14)	4.96 (2.58-8.19)	4146.87 (2108.05-7109.43)	3.22 (1.65-5.34)	-1.02 (-1.79--0.24)
Japan	6620.41 (3423.42-10692.94)	3.84 (1.99-6.19)	5487.73 (2790.24-9296.91)	2.01 (1.05-3.33)	-1.59 (-2.38--0.79)
Mexico	1508.25 (800.9-2455.14)	3.62 (1.95-5.89)	2585.43 (1327.31-4318.63)	2.02 (1.04-3.38)	-0.87 (-1.47--0.26)
Republic of Korea	1330.9 (692.71-2176.75)	4.09 (2.14-6.53)	3146.02 (1698.84-5182.92)	3.56 (1.9-5.88)	0.99 (0.56-1.41)
Russian Federation	5211.98 (2728.7-8216.78)	2.84 (1.49-4.49)	7921.12 (4113.13-12671.1)	3.41 (1.79-5.49)	1.14 (0.63-1.66)
Saudi Arabia	35.99 (16.94-62.96)	0.55 (0.26-0.95)	208.28 (96.93-356.8)	0.83 (0.41-1.38)	2.06 (1.1-3.03)
South Africa	549.12 (283.69-886.54)	2.71 (1.39-4.38)	692.85 (354.8-1134.5)	1.43 (0.73-2.38)	-1.43 (-2.02--0.84)
Turkey	594.58 (305.42-982.7)	1.68 (0.87-2.77)	1145.01 (625.86-1874.07)	1.21 (0.66-1.95)	-0.43 (-0.8--0.06)
United Kingdom	3844.92 (2015.34-6517.1)	4.35 (2.3-7.22)	3471.66 (1825.64-5803.72)	2.87 (1.48-4.72)	-1.37 (-2.02--0.72)

United States of America	13730.99 (7055-22769.43)	4.43 (2.29-7.29)	21117.84 (11806.65-34408.19)	3.71 (2.05-6.01)	0 (-0.46-0.47)

Note: Data are presented as number of cases (95% uncertainty interval) and age-standardized rates per 100,000 population (95% UI). EAPC = estimated annual percentage change.

Table S3 Trends in years of life lost (YLLs) attributable to smoking-related atrial fibrillation and atrial flutter (AF/AFL) in G20 countries from 1990 to 2021, including the number of YLL cases, age-standardized YLL rates (per 100,000 population), and estimated annual percentage changes (EAPCs), based on data from the Global Burden of Disease Study 2021

	YLLs				
	Number of YLLs cases (95% UI) in 1990	The age-standardized YLLs rate/100000 (95% UI) in 1990	Number of YLLs cases (95% UI) in 2021	The age-standardized YLLs rate/100000 (95% UI) in 2021	EAPC (95% CI)
G20	72496.8 (43059.32-101327.31)	2.63 (1.56-3.73)	132463.93 (78787.59-189831.89)	2.04 (1.22-2.93)	0.09 (-0.15-0.33)
Argentina	507.45 (313.01-719.83)	1.61 (1-2.34)	748.03 (450-1101.24)	1.34 (0.81-1.96)	0.52 (0.2-0.83)
Australia	560.19 (342.08-810.62)	2.91 (1.78-4.22)	784.34 (450.91-1187.44)	1.67 (0.98-2.49)	-0.9 (-1.46--0.34)
Brazil	2777.94 (1734.13-3935.67)	3.62 (2.21-5.22)	5020.35 (2968.13-7408.13)	2.04 (1.2-3)	-0.91 (-1.41--0.41)
Canada	1121.15 (687.13-1610.22)	3.49 (2.13-5.04)	1430.55 (850.67-2143.73)	1.89 (1.14-2.8)	-1.28 (-1.9--0.66)
China	17981.87 (10361.87-27337.68)	3.04 (1.71-4.7)	42267.04 (23274.84-64014.8)	2.24 (1.24-3.37)	0.54 (0-1.09)
European Union	18600.84 (11454.58-26530.62)	3.15 (1.94-4.48)	23338.95 (13909.81-34145.86)	2.32 (1.41-3.33)	0.12 (-0.67-0.91)

France	2386.64 (1456.58-3509.34)	2.83 (1.74-4.1)	2883.2 (1660.05-4286.66)	1.99 (1.19-2.86)	-0.37 (-1.16-0.41)
Germany	4624.02 (2719.92-6772.48)	3.63 (2.14-5.26)	6756.38 (3977.21-10036.46)	3.24 (1.95-4.71)	1.13 (0.19-2.08)
India	6257.84 (2995.8-10217.89)	1.62 (0.77-2.65)	17054.94 (9621.34-25312.39)	1.66 (0.93-2.47)	1.01 (0.17-1.86)
Indonesia	1823.9 (1095.94-2670.7)	2.29 (1.31-3.51)	5991.87 (3473.26-8937.17)	3.47 (1.96-5.38)	1.83 (0.88-2.8)
Italy	2048.14 (1222.76-2914.11)	2.4 (1.43-3.4)	2555.25 (1484.42-3794.96)	1.62 (0.98-2.37)	0.02 (-0.95-1)
Japan	4768.98 (3042.43-6687.78)	2.87 (1.83-4.04)	5110.16 (2999.63-7439.96)	1.45 (0.86-2.04)	-1.99 (-2.97--1)
Mexico	1212.66 (745.39-1724.64)	3.54 (2.13-5.11)	2011.45 (1208.22-2911.93)	1.66 (1-2.43)	-1.3 (-2.05--0.54)
Republic of Korea	816.82 (482.32-1265.96)	3.07 (1.78-4.91)	1956.86 (1065.95-3012.2)	2.11 (1.14-3.25)	0.55 (0.11-1)
Russian Federation	2637.8 (1467.42-3956.98)	1.49 (0.83-2.25)	5064.46 (3183.99-7176.62)	2.11 (1.34-2.97)	1.64 (1.13-2.16)
Saudi Arabia	30.54 (16.9-48.87)	0.51 (0.28-0.83)	162.52 (83.58-256.19)	0.67 (0.35-1.07)	1.83 (0.77-2.91)
South Africa	445.52 (268.81-641.69)	2.23 (1.33-3.3)	694.3 (408.72-1013.61)	1.51 (0.87-2.22)	-0.79 (-1.43--0.15)
Turkey	667.37 (363.06-1062.4)	2.03 (1.1-3.25)	1180.02 (671.01-1783.75)	1.32 (0.75-2.01)	-0.69 (-1.17--0.2)
United Kingdom	4091.71 (2523.99-5865.65)	4.39 (2.72-6.26)	3905.4 (2295.1-5989.88)	2.75 (1.64-4.15)	-1.04 (-1.85--0.22)

United States of America	8194.22 (5010.6-11954.85)	2.57 (1.57-3.74)	15742.71 (9243.79-23035.51)	2.67 (1.59-3.87)	0.61 (0.09-1.14)

5 Note: Data are presented as number of cases (95% uncertainty interval) and age-standardized rates per 100,000 population (95% UI). EAPC = estimated annual percentage change.

Table S4 Trends in deaths attributable to smoking-related atrial fibrillation and atrial flutter (AF/AFL) stratified by sex and age group in G20 countries from 1990 to 2021, including the number of deaths, age-standardized death rates (per 100,000 population), and estimated annual percentage changes (EAPCs), based on data from the Global Burden of Disease Study 2021

	Deaths				
	Number of Deaths cases (95% UI) in 1990	The age-standardized Deaths rate/100000 (95% UI) in 1990	Number of Deaths cases (95% UI) in 2021	The age-standardized Deaths rate/100000 (95% UI) in 2021	EAPC (95% CI)
Sex					
Both	3910.44 (2303.89-5614.18)	0.16 (0.09-0.23)	8102.87 (4749.03-11785.58)	0.13 (0.07-0.18)	-0.79 (-0.84--0.75)
Female	1279.96 (746.42-1888.8)	0.09 (0.05-0.13)	2337.25 (1311.49-3528.4)	0.06 (0.03-0.09)	-1.3 (-1.42--1.18)
Male	2630.48 (1534.11-3733.24)	0.28 (0.16-0.4)	5765.62 (3422.9-8370.89)	0.22 (0.13-0.32)	-0.74 (-0.78--0.7)
Age					
30-34 years	7.57 (4.7-10.47)	0 (0-0)	7.89 (4.83-11.16)	0 (0-0)	-0.81 (-0.94--0.69)
35-39 years	11.14 (7.11-15.27)	0 (0-0.01)	11.76 (7.27-16.52)	0 (0-0)	-0.97 (-1.06--0.88)
40-44 years	31.06 (19.29-42.4)	0.01 (0.01-0.02)	34.99 (22.12-47.77)	0.01 (0.01-0.01)	-1.31 (-1.43--1.18)

45-49 years	64.07 (39.58-88.46)	0.04 (0.02-0.05)	82.69 (50.49-115.54)	0.03 (0.02-0.04)	-1.27 (-1.41--1.13)
50-54 years	131.14 (80.95-180.86)	0.08 (0.05-0.11)	186 (112.7-259.87)	0.06 (0.04-0.08)	-1.15 (-1.25--1.05)
55-59 years	212.42 (132.41-290.53)	0.15 (0.09-0.21)	336.85 (206.14-460.22)	0.12 (0.07-0.16)	-0.75 (-0.8--0.69)
60-64 years	305.79 (189.15-420.66)	0.25 (0.15-0.34)	441.99 (272.63-611.04)	0.19 (0.12-0.26)	-0.84 (-0.9--0.79)
65-69 years	371.63 (225.98-514)	0.39 (0.24-0.54)	602.21 (366.48-844.14)	0.28 (0.17-0.4)	-0.97 (-1.02--0.92)
70-74 years	479.62 (281.54-674.12)	0.73 (0.43-1.03)	913.09 (540.94-1316.27)	0.56 (0.33-0.81)	-0.9 (-0.94--0.86)
75-79 years	608.45 (354.46-874.36)	1.25 (0.73-1.79)	1050.43 (609.35-1530.53)	1.01 (0.59-1.47)	-0.72 (-0.75--0.69)
80-84 years	696.82 (392.29-1054.89)	2.47 (1.39-3.74)	1430.7 (823.09-2175.26)	2.05 (1.18-3.11)	-0.65 (-0.72--0.58)
85-89 years	567.75 (314.29-882.56)	4.75 (2.63-7.38)	1464.07 (796.88-2244.44)	3.93 (2.14-6.03)	-0.68 (-0.77--0.58)
90-94 years	332.43 (183.86-516.01)	9.77 (5.4-15.16)	1147.49 (607-1759.51)	7.77 (4.11-11.91)	-0.82 (-0.9--0.75)
95+ years	90.55 (48.68-139.89)	11.44 (6.15-17.67)	392.7 (204.92-611.56)	8.81 (4.6-13.72)	-0.97 (-1.11--0.83)

Note: Data are presented as number of cases (95% uncertainty interval) and age-standardized rates per 100,000 population (95% UI). EAPC = estimated annual percentage change.

Table S5 Trends in years lived with disability (YLDs) attributable to smoking-related atrial fibrillation and atrial flutter (AF/AFL) stratified by sex and age group in G20 countries from 1990 to 2021, including the number of YLD cases, age-standardized YLD rates (per 100,000 population), and estimated annual percentage changes (EAPCs), based on data from the Global Burden of Disease Study 2021

	YLDs				
	Number of YLDs cases (95% UI) in 1990	The age-standardized YLDs rate/100000 (95% UI) in 1990	Number of YLDs cases (95% UI) in 2021	The age-standardized YLDs rate/100000 (95% UI) in 2021	EAPC (95% CI)
Sex					
Both	108216.23 (57638.88-176505.38)	3.54 (1.88-5.74)	189297.95 (102821.7-303660.85)	2.88 (1.57-4.61)	-0.64 (-0.67--0.6)
Female	21438.01 (11122.79-36537.25)	1.31 (0.68-2.24)	32710.38 (17423.88-54113.5)	0.93 (0.5-1.53)	-1.16 (-1.2--1.12)
Male	86778.21 (45975.51-140172.92)	6.23 (3.31-10.11)	156587.58 (84876.54-252304.66)	5.06 (2.75-8.12)	-0.62 (-0.66--0.58)
Age					
30-34 years	353.59 (148.14-717.27)	0.13 (0.05-0.26)	368.13 (153.33-701.7)	0.1 (0.04-0.18)	-0.91 (-0.95--0.86)
35-39 years	2100.33 (869.73-4279.67)	0.8 (0.33-1.64)	2216.52 (981.81-4327.31)	0.61 (0.27-1.19)	-0.98 (-1.08--0.88)
40-44 years	4681.41 (2178.32-8867.32)	2.17 (1.01-4.12)	5452.04 (2600.66-10019.91)	1.65 (0.79-3.04)	-0.86 (-1--0.71)
45-49 years	7012.48 (3421.89-12405.75)	4.06 (1.98-7.18)	10589.42 (5269.25-18542.52)	3.22 (1.6-5.64)	-0.67 (-0.81--0.52)

50-54 years	10539.37 (5062.61-17994.69)	6.62 (3.18-11.31)	17874.7 (8469.31-30470.12)	5.57 (2.64-9.49)	-0.44 (-0.54--0.34)
55-59 years	13581.45 (6846.47-23113.92)	9.63 (4.85-16.39)	24798.51 (12729.3-41032.81)	8.51 (4.37-14.09)	-0.24 (-0.33--0.16)
60-64 years	16513.57 (8430.39-27995.18)	13.42 (6.85-22.75)	26738.66 (13594.02-44653.96)	11.38 (5.79-19.01)	-0.41 (-0.47--0.34)
65-69 years	17374.58 (8766.75-28801.35)	18.09 (9.13-29.99)	31476.64 (16461.85-51299.42)	14.86 (7.77-24.22)	-0.6 (-0.67--0.52)
70-74 years	14280.23 (7167.2-24414.55)	21.76 (10.92-37.21)	28301.76 (14540.26-48068.38)	17.47 (8.97-29.67)	-0.74 (-0.8--0.67)
75-79 years	11613.37 (5980.7-20148.35)	23.8 (12.26-41.29)	19853.79 (10310.36-34429.53)	19.11 (9.92-33.14)	-0.79 (-0.85--0.74)
80-84 years	6372.63 (3143.48-11878.15)	22.59 (11.14-42.1)	12337.25 (6296.22-22570.69)	17.65 (9.01-32.28)	-0.93 (-0.98--0.88)
85-89 years	2861.49 (1412.72-5151.51)	23.92 (11.81-43.05)	6540.56 (3261.89-11665.3)	17.56 (8.76-31.32)	-1.17 (-1.23--1.11)
90-94 years	784.07 (378.72-1391.09)	23.04 (11.13-40.88)	2276.9 (1121.3-3963.14)	15.41 (7.59-26.82)	-1.48 (-1.54--1.42)
95+ years	147.66 (67.25-271.39)	18.65 (8.5-34.29)	473.07 (208.91-826.29)	10.61 (4.69-18.53)	-2 (-2.07--1.92)

Note: Data are presented as number of cases (95% uncertainty interval) and age-standardized rates per 100,000 population (95% UI). EAPC = estimated annual percentage change.

Table S6 Trends in years of life lost (YLLs) attributable to smoking-related atrial fibrillation and atrial flutter (AF/AFL) stratified by sex and age group in G20 countries from 1990 to 2021, including the number of YLL cases, age-standardized YLL rates (per 100,000 population), and estimated annual percentage changes (EAPCs), based on data from the Global Burden of Disease Study 2021

	YLLs				
	Number of YLLs cases (95% UI) in 1990	The age-standardized YLLs rate/100000 (95% UI) in 1990	Number of YLLs cases (95% UI) in 2021	The age-standardized YLLs rate/100000 (95% UI) in 2021	EAPC (95% CI)
Sex					
Both	72496.8 (43059.32-101327.31)	2.63 (1.56-3.73)	132463.93 (78787.59-189831.89)	2.04 (1.22-2.93)	-0.84 (-0.88--0.81)
Female	20323.65 (11926.49-29623.22)	1.33 (0.78-1.93)	33145.85 (19203.93-49198.69)	0.9 (0.52-1.33)	-1.28 (-1.37--1.18)
Male	52173.15 (31087.17-72611.28)	4.46 (2.59-6.3)	99318.08 (59637.73-141844.07)	3.5 (2.1-5.02)	-0.8 (-0.83--0.77)
Age					
30-34 years	436.86 (271.37-604.19)	0.16 (0.1-0.22)	455.49 (278.9-644.09)	0.12 (0.07-0.17)	-0.82 (-0.94--0.69)
35-39 years	587.93 (375.46-805.57)	0.23 (0.14-0.31)	620.45 (383.72-871.83)	0.17 (0.11-0.24)	-0.97 (-1.07--0.88)
40-44 years	1487.02 (923.36-2029.56)	0.69 (0.43-0.94)	1674.09 (1057.99-2285.06)	0.51 (0.32-0.69)	-1.31 (-1.43--1.19)
45-49 years	2749.72 (1698.77-3795.76)	1.59 (0.98-2.2)	3549.94 (2167.29-4959.8)	1.08 (0.66-1.51)	-1.28 (-1.42--1.13)

50-54 years	5004.08 (3089.21-6901.29)	3.15 (1.94-4.34)	7099.14 (4301.69-9918.92)	2.21 (1.34-3.09)	-1.15 (-1.26--1.05)
55-59 years	7109.45 (4431.07-9724.17)	5.04 (3.14-6.9)	11285.52 (6905.96-15419.12)	3.87 (2.37-5.29)	-0.75 (-0.8--0.69)
60-64 years	8824.54 (5458.29-12139.32)	7.17 (4.44-9.87)	12753.27 (7866.9-17631.63)	5.43 (3.35-7.51)	-0.84 (-0.89--0.79)
65-69 years	9033.63 (5492.94-12493.21)	9.41 (5.72-13.01)	14630.85 (8904.58-20509.7)	6.91 (4.2-9.68)	-0.97 (-1.02--0.91)
70-74 years	9588.62 (5628.16-13478.69)	14.61 (8.58-20.54)	18237.76 (10806.43-26290.83)	11.26 (6.67-16.23)	-0.91 (-0.94--0.87)
75-79 years	9708.62 (5654.63-13948.5)	19.9 (11.59-28.58)	16758.2 (9720.16-24411.29)	16.13 (9.36-23.5)	-0.73 (-0.76--0.7)
80-84 years	8718.82 (4907.56-13200.23)	30.9 (17.4-46.79)	17831.48 (10255.94-27109.12)	25.51 (14.67-38.78)	-0.66 (-0.73--0.59)
85-89 years	5642.17 (3122.69-8769.76)	47.15 (26.1-73.29)	14484.67 (7883.69-22204.76)	38.89 (21.16-59.61)	-0.69 (-0.78--0.6)
90-94 years	2867.01 (1585.65-4450.14)	84.25 (46.59-130.77)	9892 (5232.74-15166.82)	66.95 (35.42-102.65)	-0.83 (-0.9--0.75)
95+ years	738.34 (396.89-1140.03)	93.28 (50.14-144.02)	3191.06 (1665.9-4971.95)	71.57 (37.36-111.5)	-0.99 (-1.13--0.85)

Note: Data are presented as number of cases (95% uncertainty interval) and age-standardized rates per 100,000 population (95% UI). EAPC = estimated annual percentage change

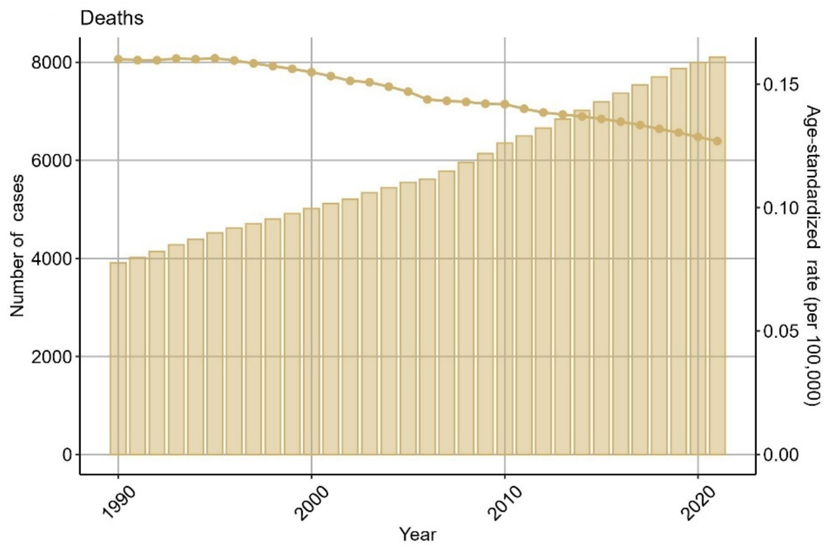

Figure S1. Trends in deaths and age-standardized mortality rates of atrial fibrillation and atrial flutter attributable to smoking in G20 countries, 1990 - 2021.

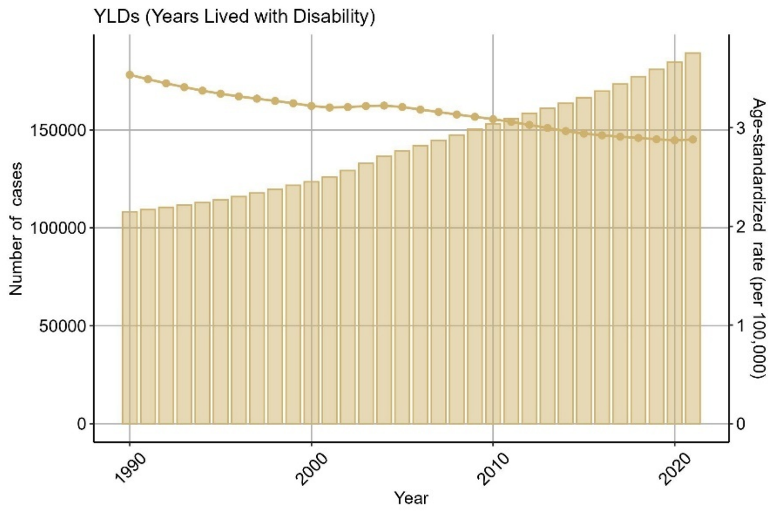

Figure S2. Trends in years lived with disability (YLDs) and age-standardized YLD rates of atrial fibrillation and atrial flutter attributable to smoking in G20 countries, 1990 – 2021.

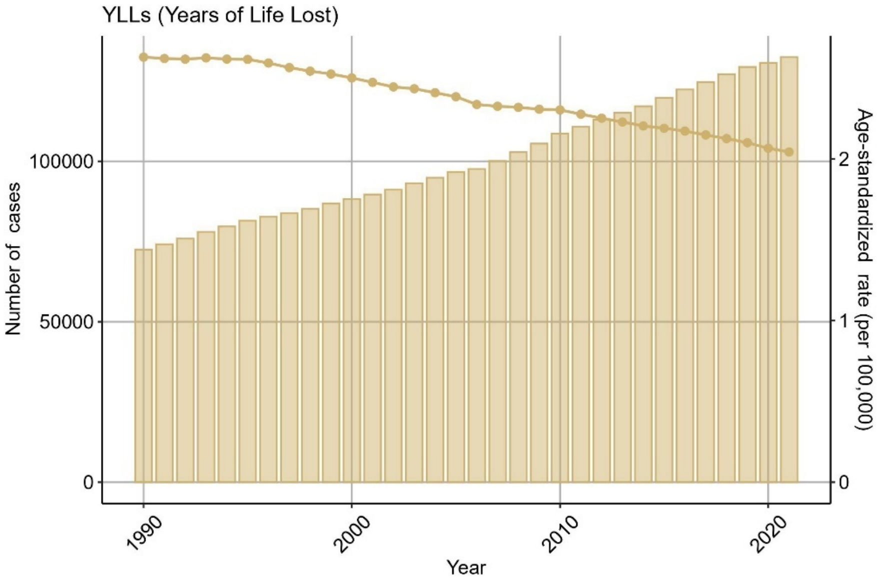

Figure S3. Trends in years of life lost (YLLs) and age-standardized YLL rates of atrial fibrillation and atrial flutter attributable to smoking in G20 countries, 1990–2021.

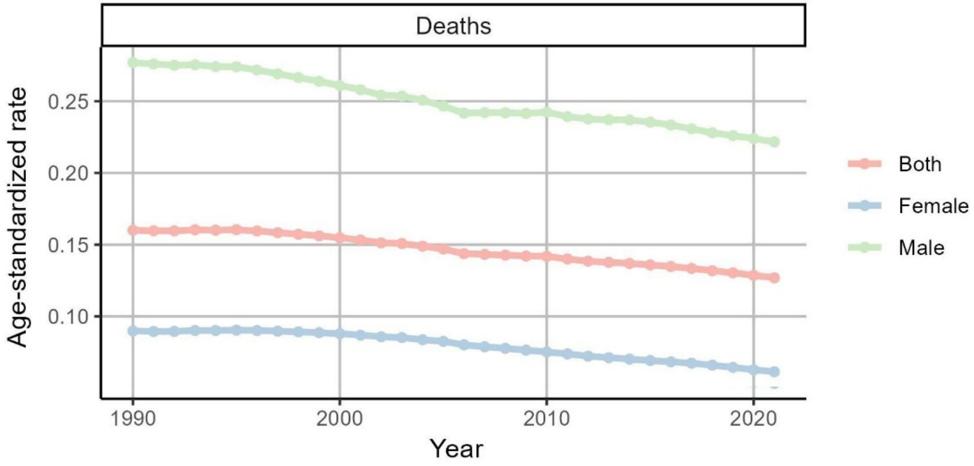

Figure S4. Trends in age-standardized mortality rates of atrial fibrillation and atrial flutter attributable to smoking in G20 countries by sex, 1990 - 2021.

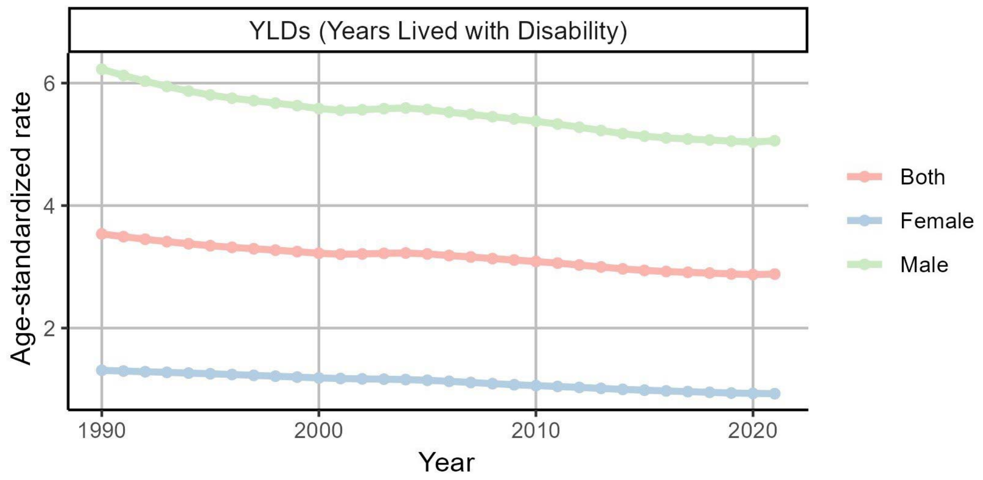

Figure S5. Trends in age-standardized YLD rates of atrial fibrillation and atrial flutter attributable to smoking in G20 countries by sex, 1990–2021.

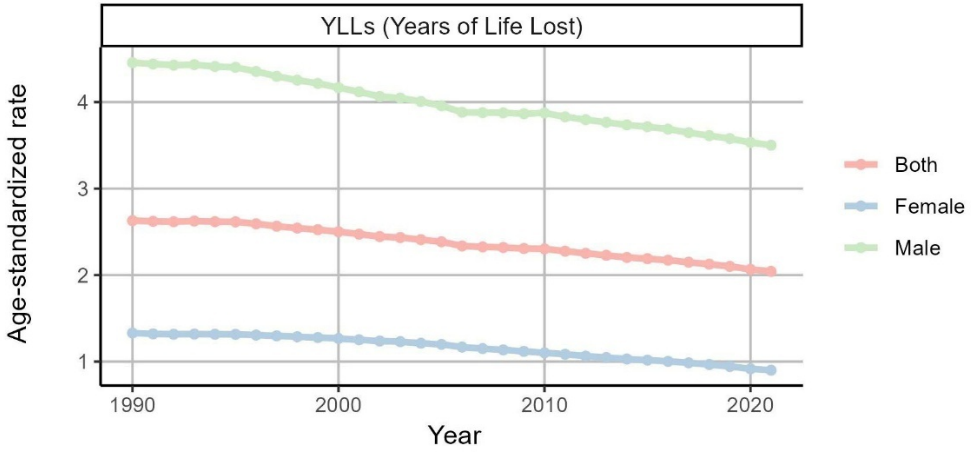

Figure S6. Trends in age-standardized YLL rates of atrial fibrillation and atrial flutter attributable to smoking in G20 countries by sex, 1990–2021.

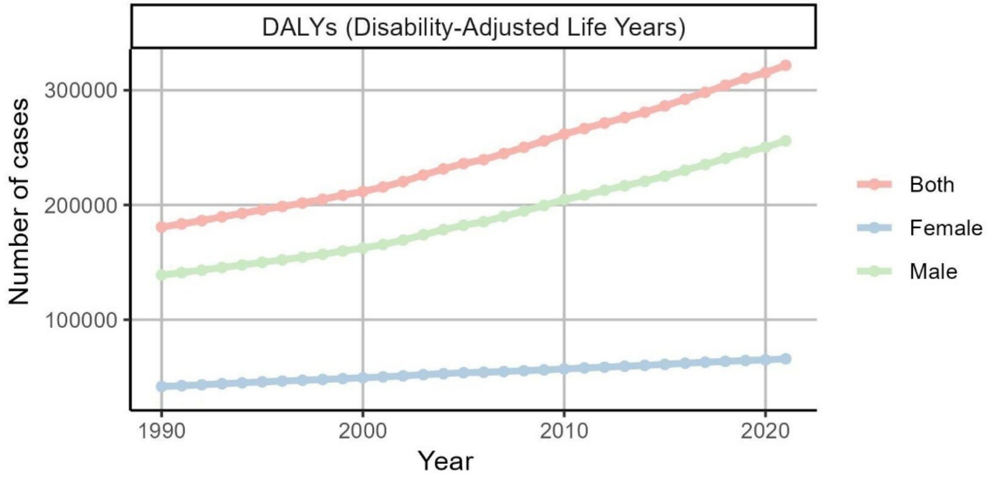

Figure S7. Trends in the number of DALYs attributable to smoking-related atrial fibrillation and atrial flutter in G20 countries by sex, 1990–2021.

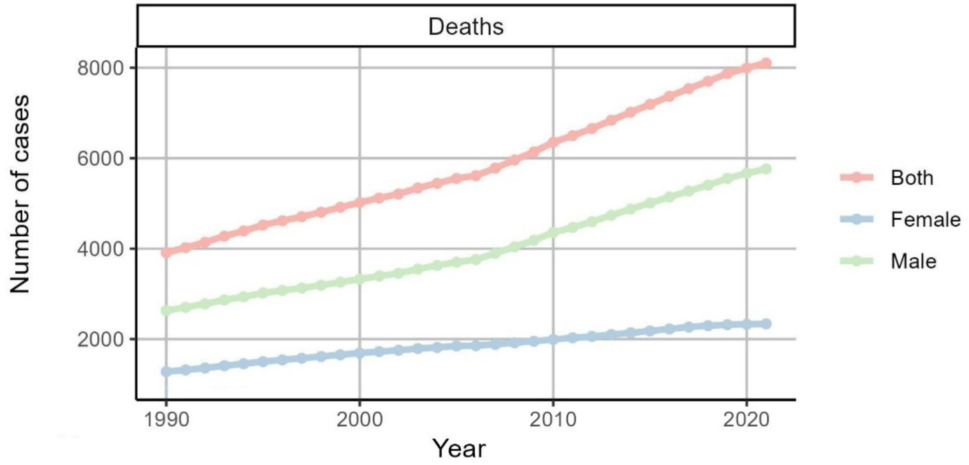

Figure S8. Trends in the number of deaths attributable to smoking-related atrial fibrillation and atrial flutter in G20 countries by sex, 1990–2021.

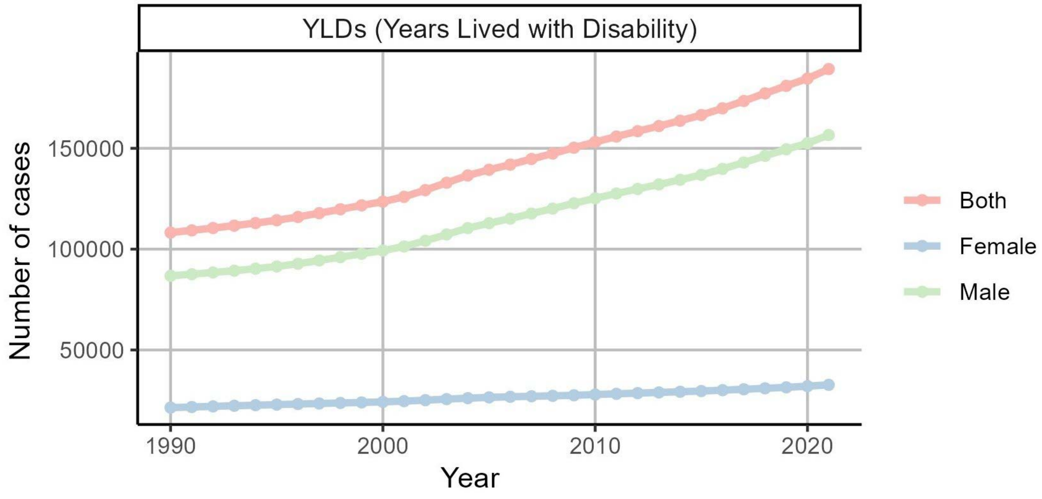

Figure S9. Trends in the number of YLDs attributable to smoking-related atrial fibrillation and atrial flutter in G20 countries by sex, 1990–2021.

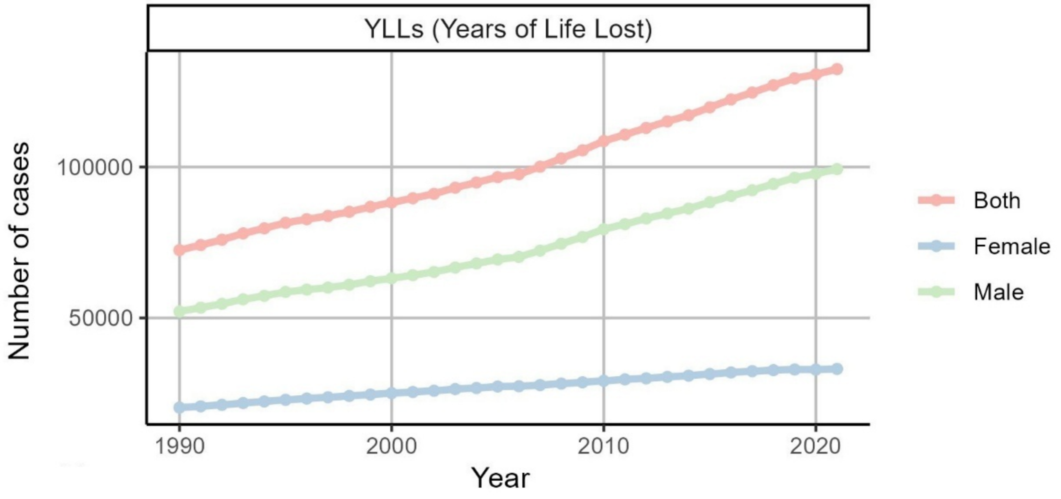

Figure S10. Trends in the number of YLLs attributable to smoking-related atrial fibrillation and atrial flutter in G20 countries by sex, 1990–2021.

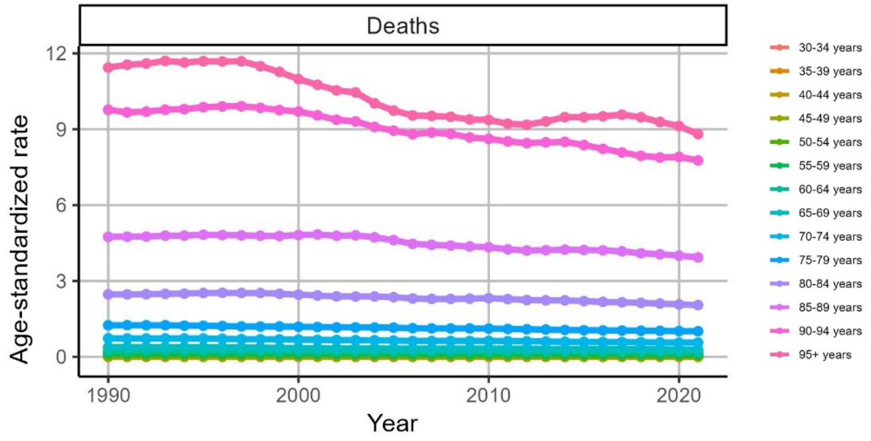

Figure S11. Trends in age-standardized mortality rates of atrial fibrillation and atrial flutter attributable to smoking across age groups in G20 countries, 1990–2021.

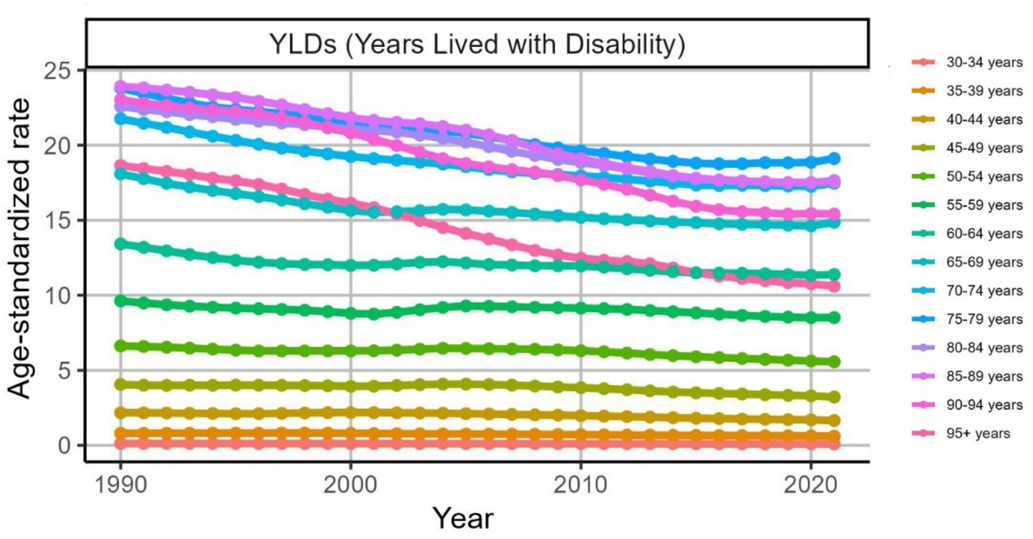

Figure S12. Trends in age-standardized YLD rates of atrial fibrillation and atrial flutter attributable to smoking across age groups in G20 countries, 1990–2021.

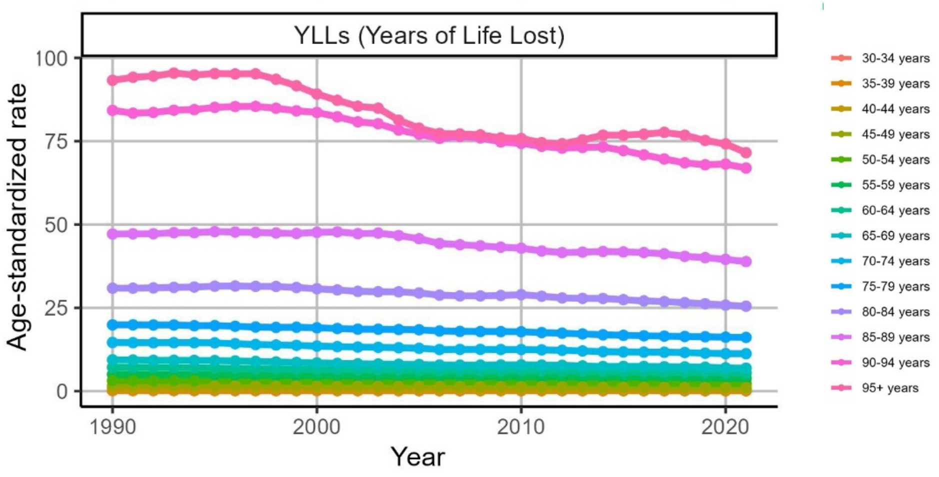

Figure S13. Trends in age-standardized YLL rates of atrial fibrillation and atrial flutter attributable to smoking across age groups in G20 countries, 1990–2021.

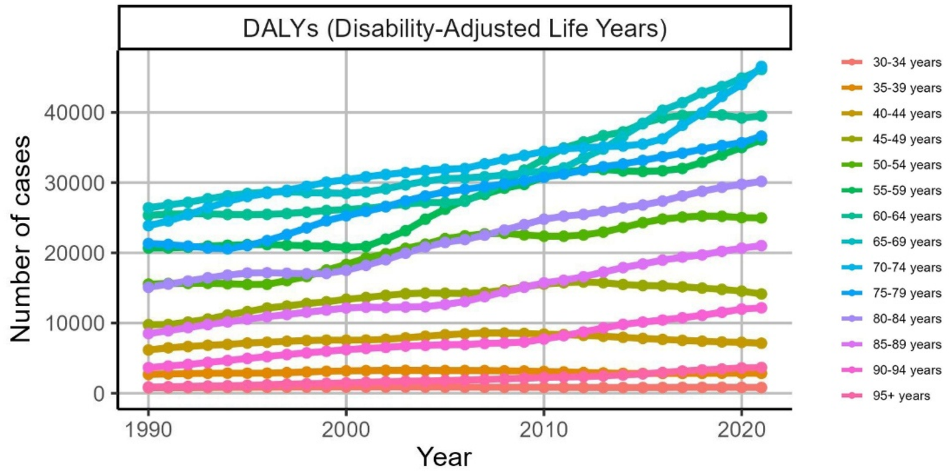

Figure S14. Trends in the number of DALYs attributable to smoking-related atrial fibrillation and atrial flutter across age groups in G20 countries, 1990–2021.

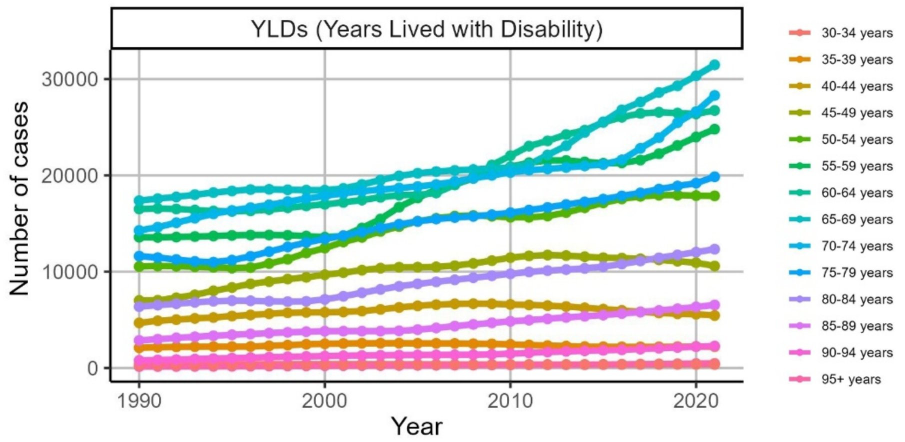

Figure S15. Trends in the number of YLDs attributable to smoking-related atrial fibrillation and atrial flutter across age groups in G20 countries, 1990–2021.

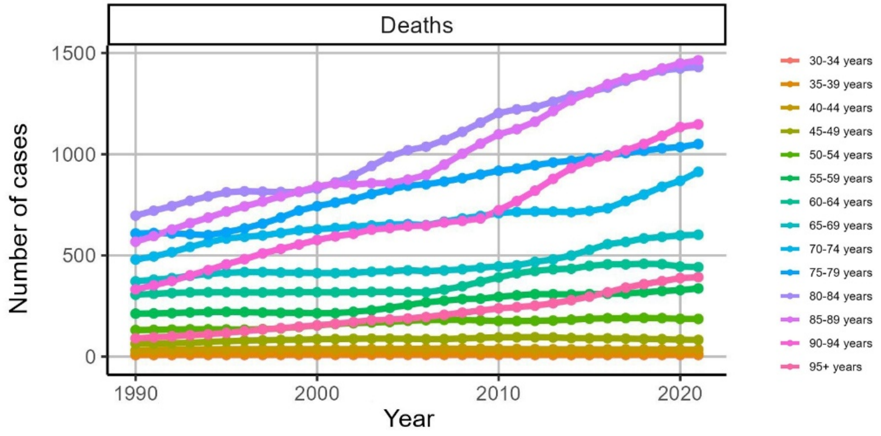

Figure S16. Trends in the number of deaths attributable to smoking-related atrial fibrillation and atrial flutter across age groups in G20 countries, 1990–2021.

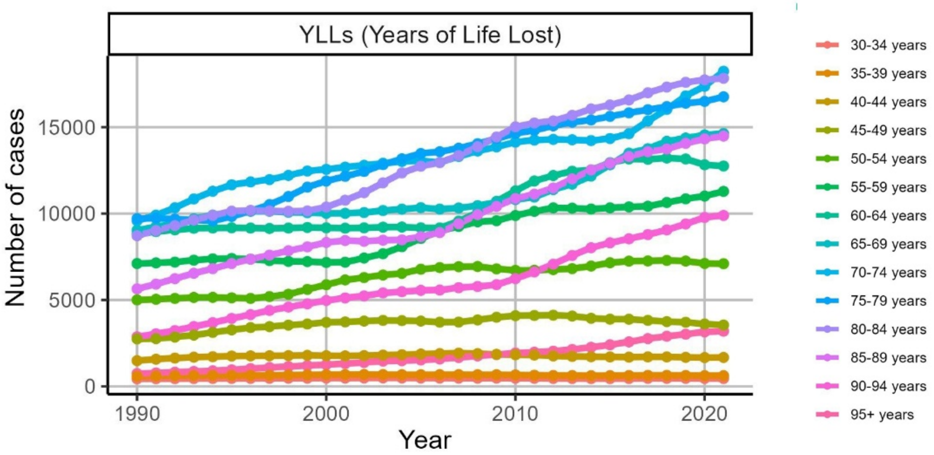

Figure S17. Trends in the number of YLLs attributable to smoking-related atrial fibrillation and atrial flutter across age groups in G20 countries, 1990–2021.

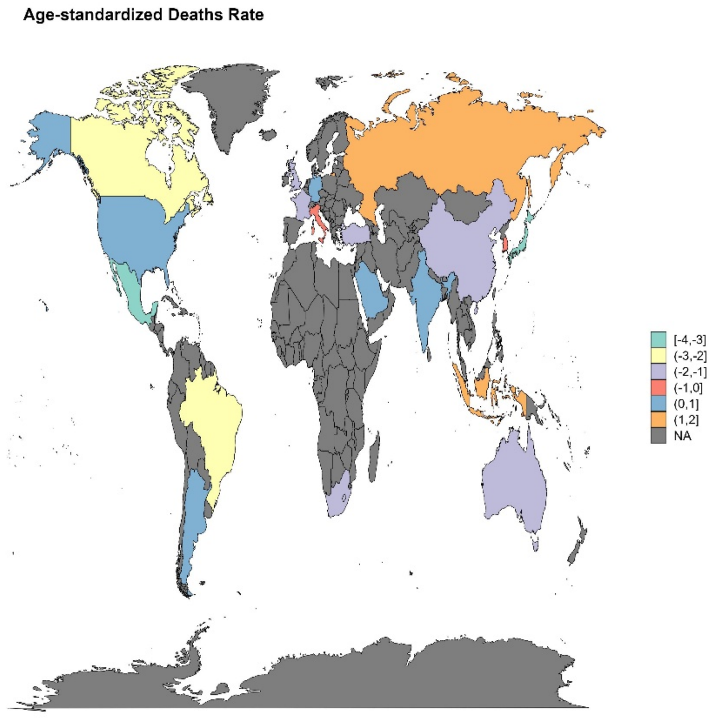

Figure S18. Global distribution of age-standardized mortality rates of atrial fibrillation and atrial flutter attributable to smoking, 2021.

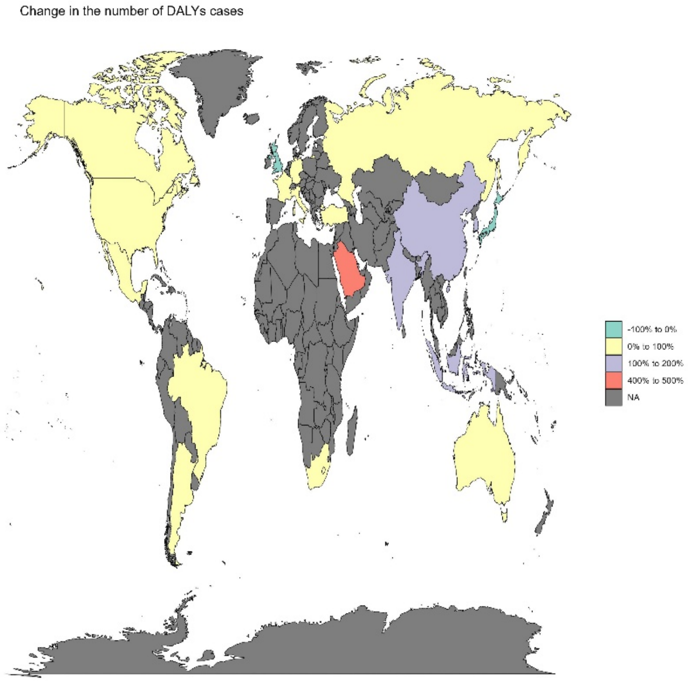

Figure S19. Percentage change in the number of DALYs attributable to smoking-related atrial fibrillation and atrial flutter by country, 1990–2021.

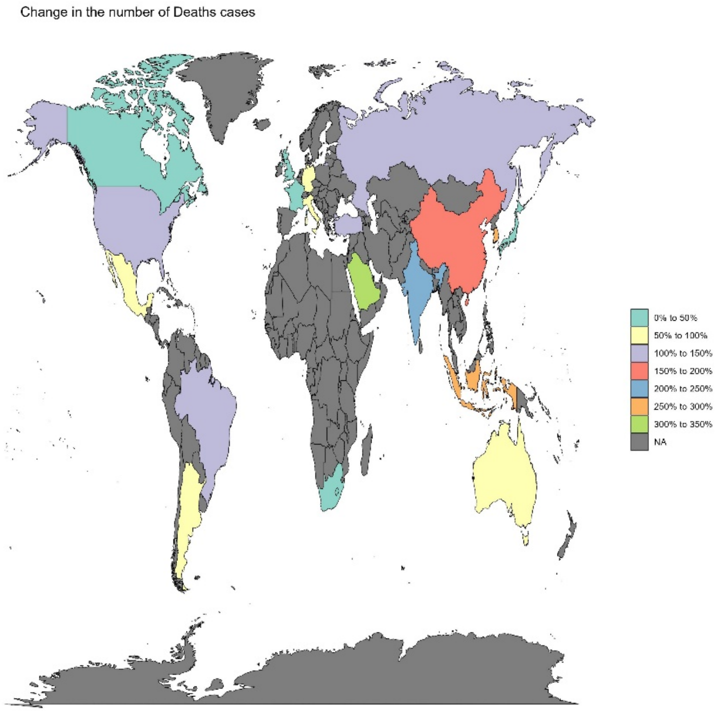

Figure S20. Percentage change in the number of deaths attributable to smoking-related atrial fibrillation and atrial flutter by country, 1990–2021.

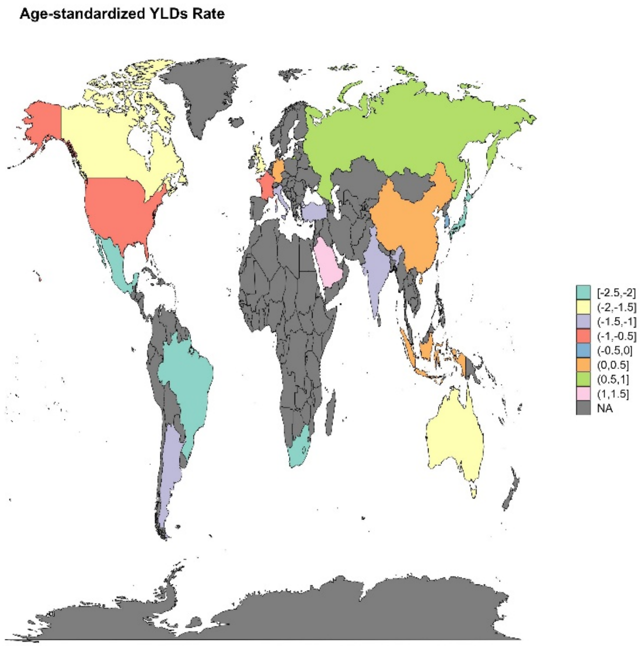

Figure S21. Global distribution of age-standardized YLD rates of atrial fibrillation and atrial flutter attributable to smoking, 2021.

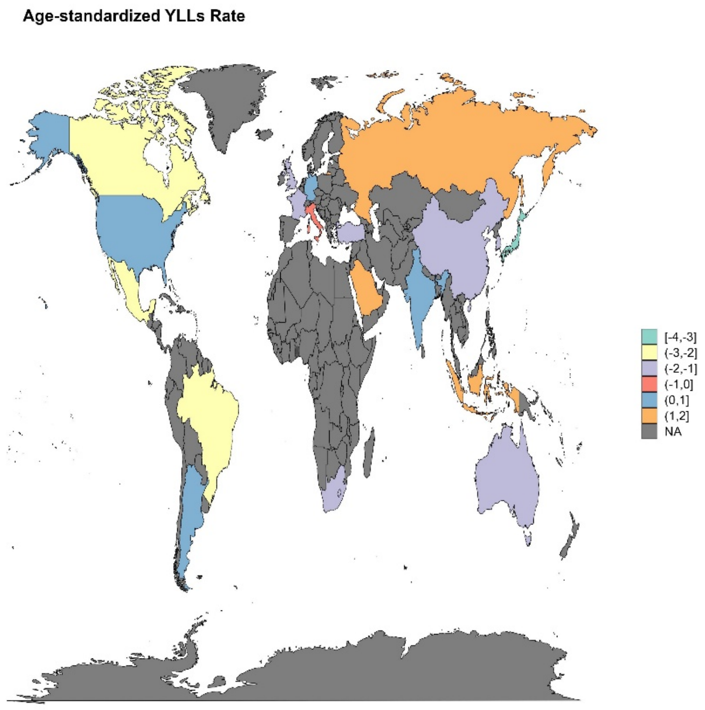

Figure S22. Global distribution of age-standardized YLL rates of atrial fibrillation and atrial flutter attributable to smoking, 2021.

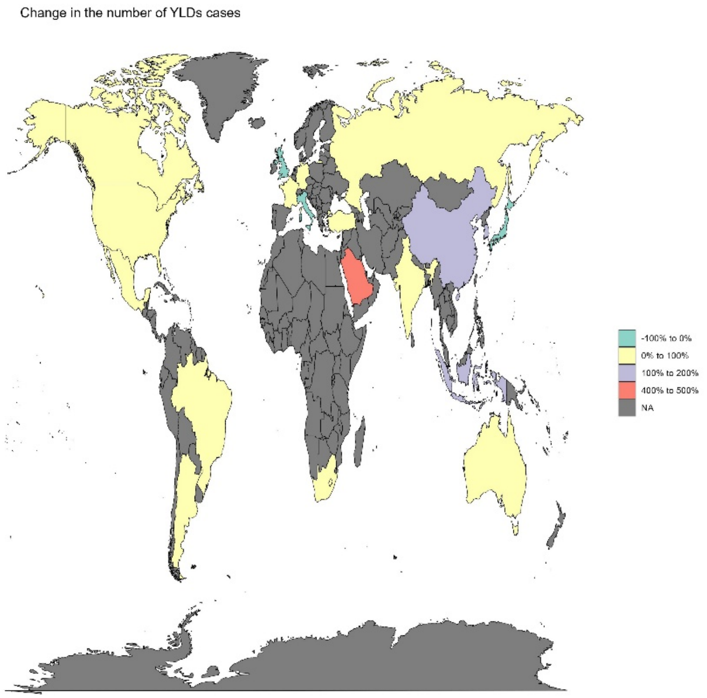

Figure S23. Percentage change in the number of YLDs attributable to smoking-related atrial fibrillation and atrial flutter by country, 1990–2021.

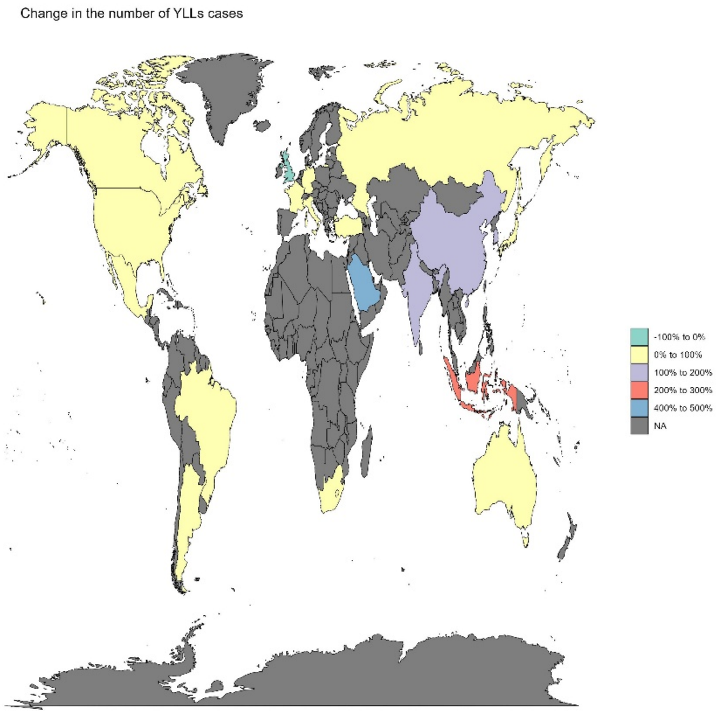

Figure S24. Percentage change in the number of YLLs attributable to smoking-related atrial fibrillation and atrial flutter by country, 1990–2021.

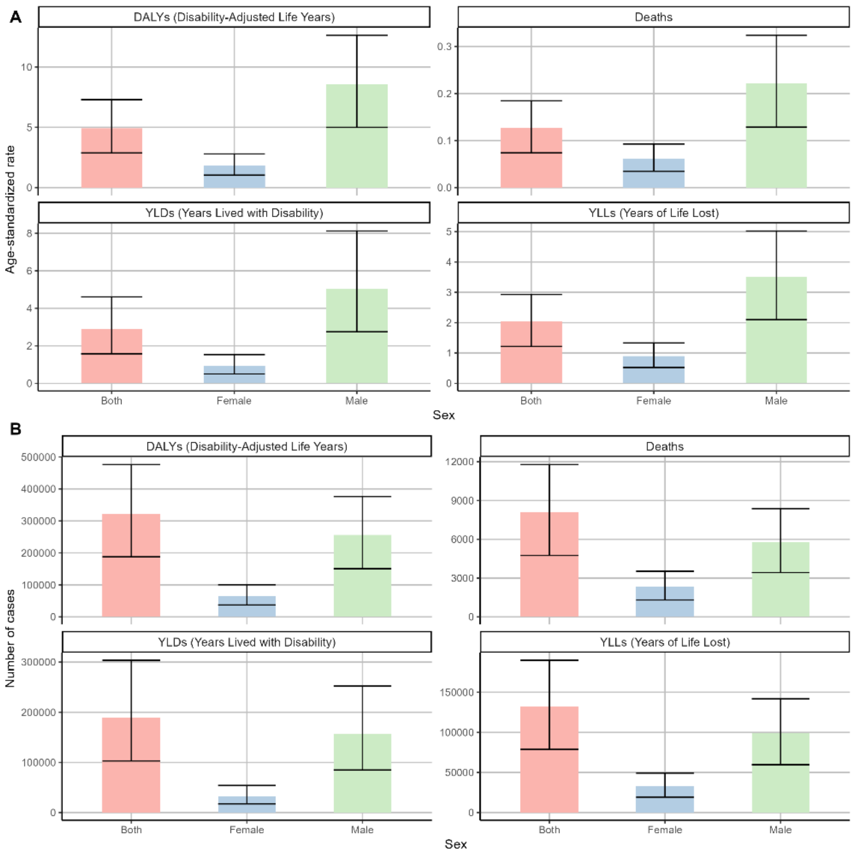

Figure S25. The number of cases and ASRs of Deaths, YLDs, YLLs and DALYs related to AF/AFL caused by smoking within the G20 in 2021. (A): ASR; (B): Number of cases. Error bars indicate 95% uncertainty intervals (UIs) derived from the Global Burden of Disease (GBD) 2021 estimates.

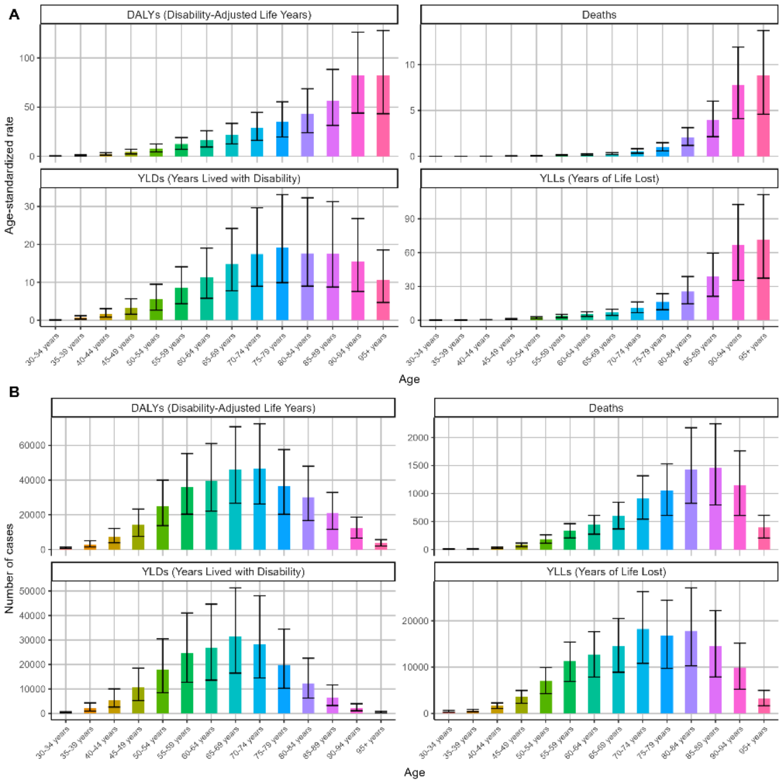

Figure S26. The number of cases and ASRs of Deaths, YLDs, YLLs and DALYs related to AF/AFL caused by smoking within the G20 in 2021 in different age group. (A): ASR; (B): Number of cases. Error bars indicate 95% uncertainty intervals (UIs) derived from the Global Burden of Disease (GBD) 2021 estimates.

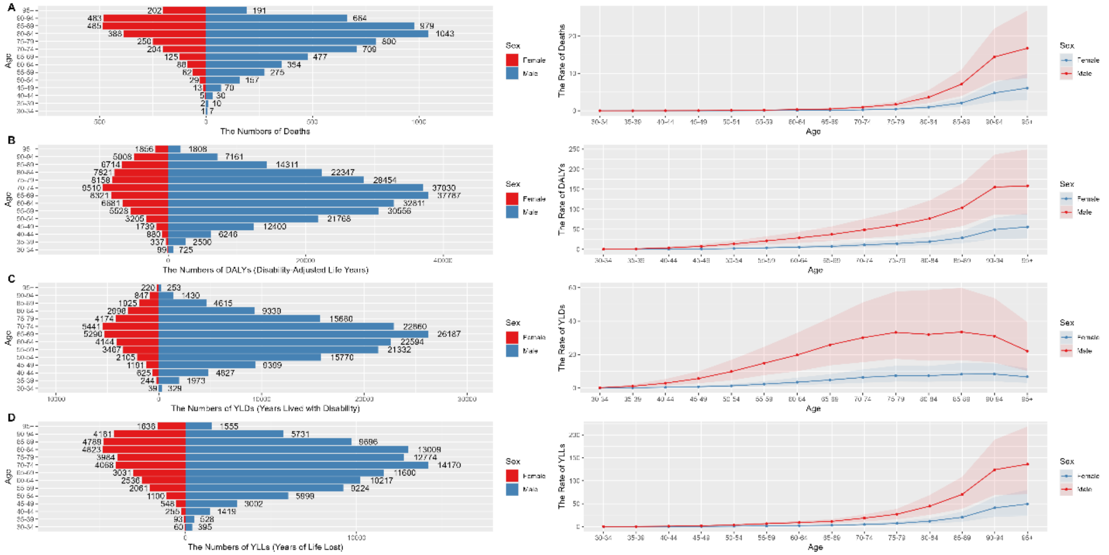

Figure S27. Sex-age trend of Deaths, YLDs, YLLs and DALYs related to AF/AFL caused by smoking within the G20 in 2021. (A): Deaths; (B): DALYs; (C): YLDs; (D): YLLs

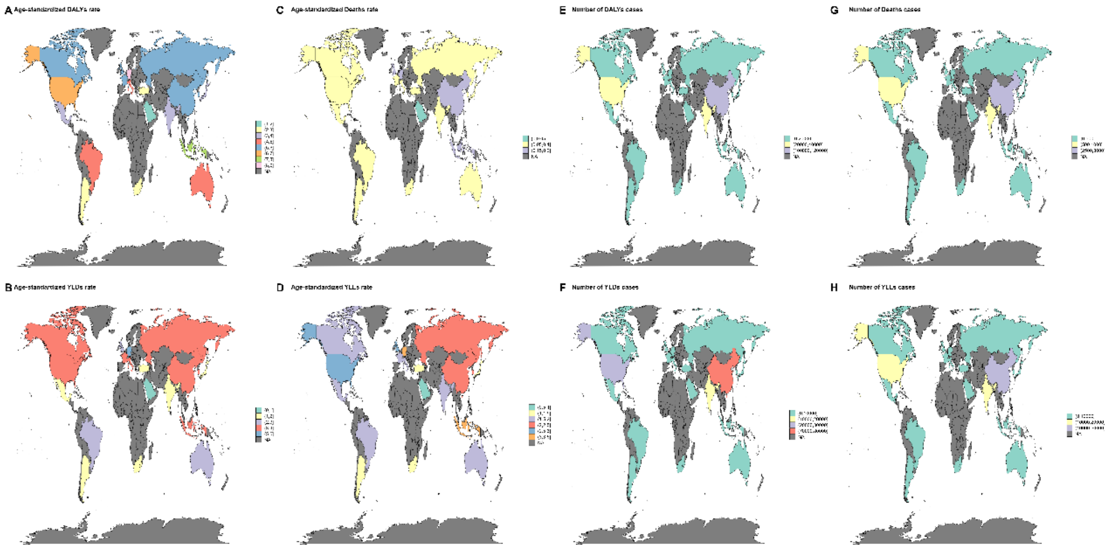

Figure S28. The number of cases and ASRs of Deaths, YLDs, YLLs and DALYs related to AF/AFL caused by smoking within the G20 countries in 2021. (A): ASR of DALYs; (B): ASR of YLDs; (C): ASR of Deaths; (D): ASR of YLLs; (E): Number of DALYs cases; (F): Number of YLDs cases; (G): Number of Deaths cases; (H): Number of YLLs cases

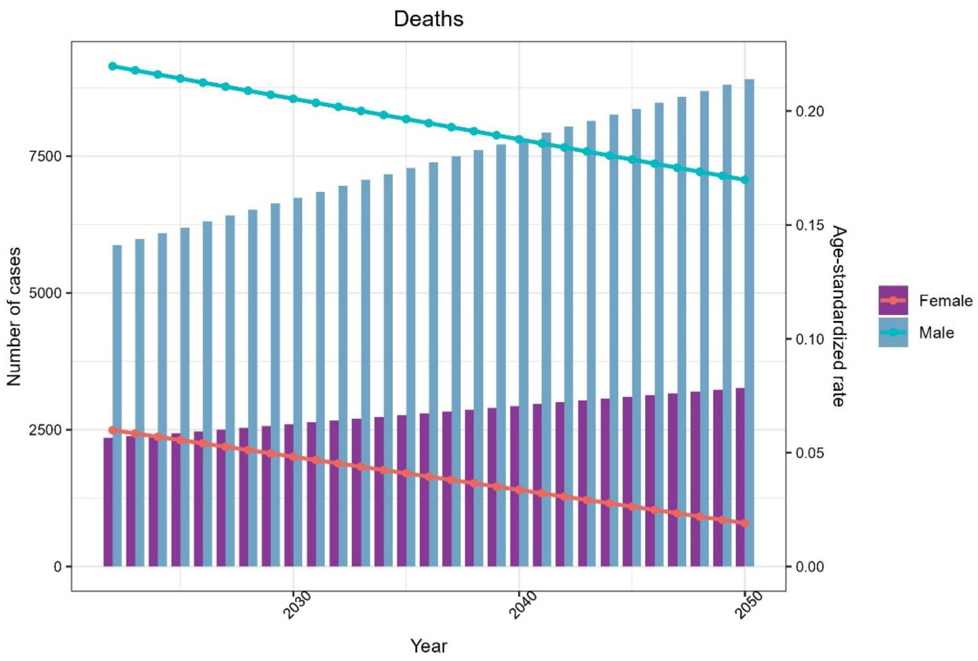

Figure S29. Projected trends in deaths and age-standardized mortality rates of atrial fibrillation and atrial flutter attributable to smoking in G20 countries by sex, 2022–2050.

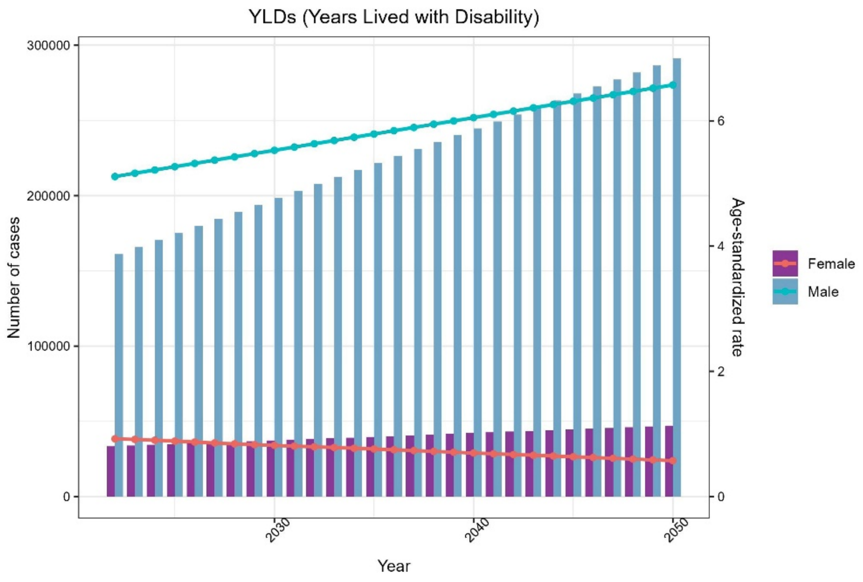

Figure S30. Projected trends in years lived with disability (YLDs) and age-standardized YLD rates of atrial fibrillation and atrial flutter attributable to smoking in G20 countries by sex, 2022–2050.

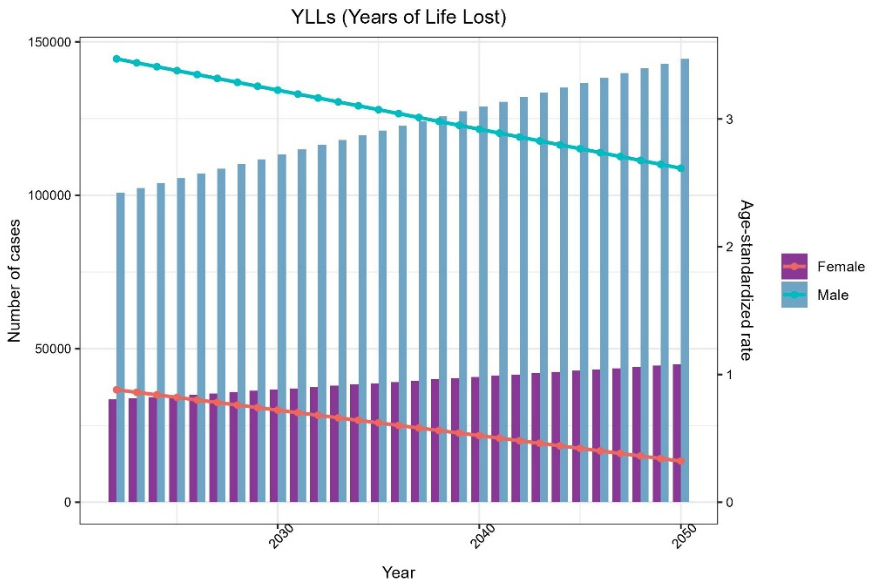

Figure S31. Projected trends in years of life lost (YLLs) and age-standardized YLL rates of atrial fibrillation and atrial flutter attributable to smoking in G20 countries by sex, 2022–2050.

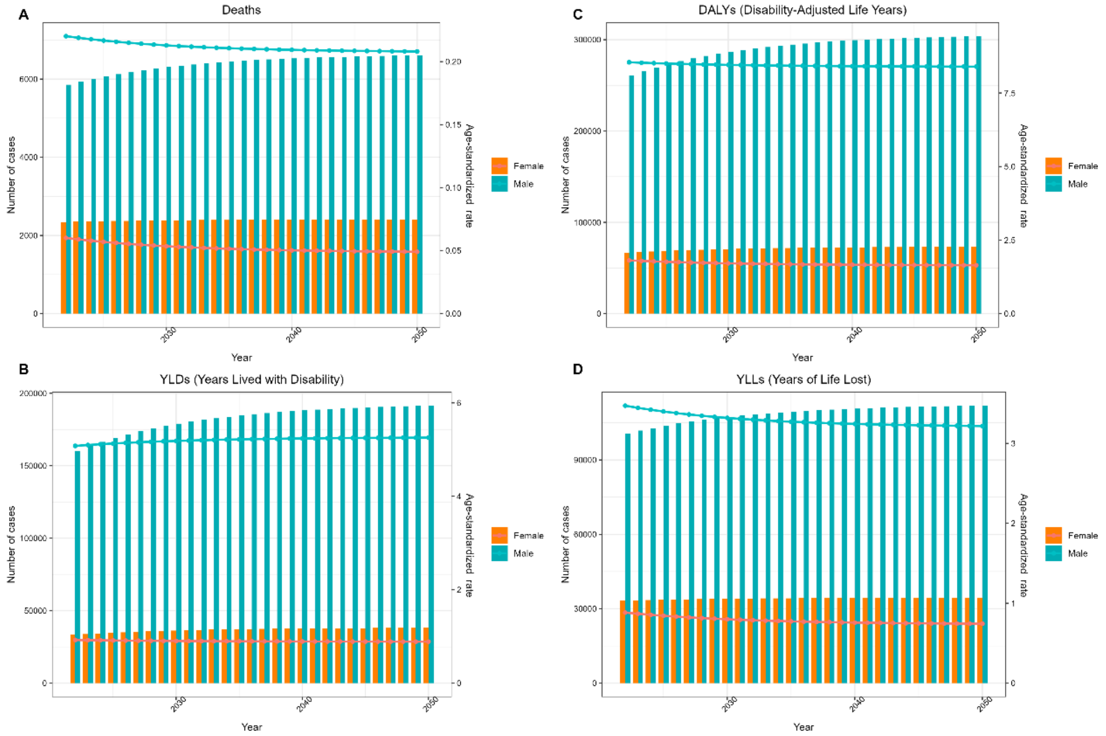

Figure S32. Prediction results of the ES model applied to predict smoking - related Deaths, YLDs, YLLs, and DALYs due to AF/AFL within G20 countries from 2022-2050. (A): Deaths; (B): YLDs; (C): DALYs; (D): YLLs

© 2026 Yang W. et al.
